# Cyanide Complexes Based on {Mo_6_I_8_}^4+^ and {W_6_I_8_}^4+^ Cluster Cores

**DOI:** 10.3390/molecules25245796

**Published:** 2020-12-08

**Authors:** Aleksei S. Pronin, Spartak S. Yarovoy, Yakov M. Gayfulin, Aleksey A. Ryadun, Konstantin A. Brylev, Denis G. Samsonenko, Ilia V. Eltsov, Yuri V. Mironov

**Affiliations:** 1 Nikolaev Institute of Inorganic Chemistry SB RAS, 3, Acad. Lavrentiev ave., 630090 Novosibirsk, Russia; pronin@niic.nsc.ru (A.S.P.); YSS@niic.nsc.ru (S.S.Y.); ryadunalexey@mail.ru (A.A.R.); brylev@niic.nsc.ru (K.A.B.); denis@niic.nsc.ru (D.G.S.); 2 Department of Natural Sciences, Novosibirsk State University, 2, Pirogova str., 630090 Novosibirsk, Russia; eiv@fen.nsu.ru

**Keywords:** cluster compounds, molybdenum, tungsten, cyanide ligand, crystal structure, luminescence, hydrolytic stability

## Abstract

Compounds based on new cyanide cluster anions [{Mo_6_I_8_}(CN)_6_]^2–^, *trans*-[{Mo_6_I_8_}(CN)_4_(MeO)_2_]^2–^ and *trans*-[{W_6_I_8_}(CN)_2_(MeO)_4_]^2−^ were synthesized using mechanochemical or solvothermal synthesis. The crystal and electronic structures as well as spectroscopic properties of the anions were investigated. It was found that the new compounds exhibit red luminescence upon excitation by UV light in the solid state and solutions, as other cluster complexes based on {Mo_6_I_8_}^4+^ and {W_6_I_8_}^4+^ cores do. The compounds can be recrystallized from aqueous methanol solutions; besides this, it was shown using NMR and UV-Vis spectroscopy that anions did not undergo hydrolysis in the solutions for a long time. These facts indicate that hydrolytic stabilization of {Mo_6_I_8_} and {W_6_I_8_} cluster cores can be achieved by coordination of cyanide ligands.

## 1. Introduction

Chemistry and applications of compounds based on octahedral cluster cores {M_6_X_8_}^4+^ (M = Mo or W; X = Cl, Br or I) have been studied for several decades due to a number of interesting physicochemical properties, including bright luminescence in the red and near infrared regions [1,2,3,4,5,6,7] and reversible redox transformations [8,9]. High luminescence quantum yields and lifetimes in combination with tunability of chemical properties by changing ligand environments made it possible to create a number of promising luminescent and photocatalytic materials based on these clusters [10,11,12,13,14,15]. The phosphorescence of clusters is also associated with the efficient singlet oxygen production [16,17]. Therefore, compounds and materials based on {M_6_X_8_}^4+^ clusters can potentially be applied in photodynamic therapy [18,19,20,21,22], antibacterial and antiviral applications [23,24,25]. Besides this, luminescence of clusters was successfully used in bioimaging [18,26,27].

An interesting and less studied area is the use of luminescent octahedral cluster complexes of molybdenum and tungsten as building blocks for the synthesis of functional coordination polymers. High symmetry and large volume of the cluster complexes make them convenient for the design of crystalline coordination polymers and metal-organic frameworks, while the spectroscopic and redox features of the clusters can be used in order to impart the desired functionality to the solid material. The majority of coordination polymers based on octahedral metal cluster complexes known to date have been obtained based on the cyanide-coordinated clusters [28,29,30]. The presence of ambidentate apical cyanide ligands makes it possible to coordinate transition and post-transition metal cations and to obtain crystalline coordination polymers by self-assembly. Using {M_6_X_8_}-type cyanoclusters is interesting for obtaining new luminescent coordination polymers. For now, the structure of only one cyanide cluster based on an {Mo_6_X_8_}^4+^ core has been described in the literature, namely [{Mo_6_Br_8_}(CN)_6_]^2−^ [31,32], while the tungsten cyanide clusters of that type are unknown. Therefore, further progress in this field requires the development of methods for the synthesis of cyanide molybdenum and tungsten halide clusters.

In this work, we report on the synthesis of the first cyanide cluster anions based on {Mo_6_I_8_}^4+^ and {W_6_I_8_}^4+^ cores, namely [{Mo_6_I_8_}(CN)_6_]^2−^, *trans*-[{Mo_6_I_8_}(CN)_4_(MeO)_2_]^2−^ and *trans*-[{W_6_I_8_}(CN)_2_(MeO)_4_]^2−^. Their crystal structures and spectroscopic properties in the solid state and solutions were investigated together with calculated geometry and electronic structures. To obtain new compounds, two different ways of synthesis were tested, namely, substitution of terminal iodide ligands in solvothermal conditions and mechanochemical depolymerization of Mo_6_I_12_. Each way was found to be usable for obtaining target compounds in preparative amounts. The new anions have shown an outstanding stability in aqueous solutions, demonstrating the stabilizing influence of terminal cyanides.

## 2. Results and Discussion

### 2.1. Synthesis

Until now, only one structure of a cyanide cluster based on halide octahedral molybdenum core has been reported, namely [Mo_6_Br_8_(CN)_6_]^2−^ [31]. It was shown that this complex formed polymeric compounds with transition metal cations [32]. For octahedral tungsten halides, cyanide compounds have not been described. At the same time, complexes based on {Mo_6_I_8_}^4+^ and {W_6_I_8_}^4+^ cluster cores are usually very bright luminophores, which makes them interesting as components of supramolecular systems and coordination polymers.

**Scheme 1:**
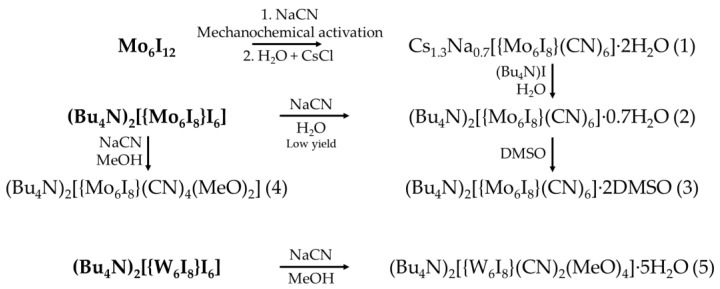
The synthetic ways of obtaining the compounds **1**–**5**.

In this work, two approaches were realized for the preparation of cyanide cluster complexes based on {Mo_6_I_8_}^4+^ and {W_6_I_8_}^4+^ cores (Scheme 1). The first approach is depolymerization of M_6_I_12_ compounds by mechanochemical activation. The reaction between polymeric Mo_6_I_12_ and NaCN in the ball mill led to the formation of water-soluble products. After filtration of the solution, addition of CsCl and evaporation, an orange microcrystalline powder was isolated, which was identified as Cs_1.3_Na_0.7_[{Mo_6_I_8_}(CN)_6_]·2H_2_O (**1**) based on elemental analysis and EDS. The reaction of an aqueous solution of this compound with an aqueous solution of Bu_4_NI led to the precipitation of the compound (Bu_4_N)_2_[{Mo_6_I_8_}(CN)_6_]·0.7H_2_O (**2**), which is soluble in polar organic solvents and can be easily recrystallized from CH_3_CN/H_2_O or MeOH/H_2_O mixtures. Recrystallization from dimethyl sulfoxide (DMSO) led to removal of the solvate H_2_O molecules and precipitation of compound **3**. The yield of compound **2** is about 50% with respect to the initially taken Mo_6_I_12_, which makes it possible to obtain it in preparative amounts. It was also shown that this compound can be obtained with a very low yield in a solvothermal reaction between the salt (Bu_4_N)_2_[{Mo_6_I_8_}I_6_] and NaCN in an aqueous solution. The reaction gave an insoluble brown precipitate and a cloudy yellow solution, from which, after filtration and evaporation, several crystals of compound **2** were isolated. An attempt to obtain a tungsten analog of the [{Mo_6_I_8_}(CN)_6_]^2−^ anion by mechanochemical activation of W_6_I_12_ in the presence of NaCN was unsuccessful: the resulting brown-black product was unstable to hydrolysis. It is interesting to note that soluble trinuclear clusters of both molybdenum and tungsten, namely (Et_4_N)_2_[W_3_S_7_Br_6_], (Et_4_N)_2_[W_3_Se_7_Br_6_], and [Mo_3_Se_7_(dtc)_3_]dtc (dtc = diethyldithiocarbamate), were synthesized earlier using mechanochemical activation of polymeric solids W_3_S_7_Br_4_, W_3_Se_7_Br_4_ and Mo_3_Se_7_Br_4_, respectively [33].

**Figure 1:**
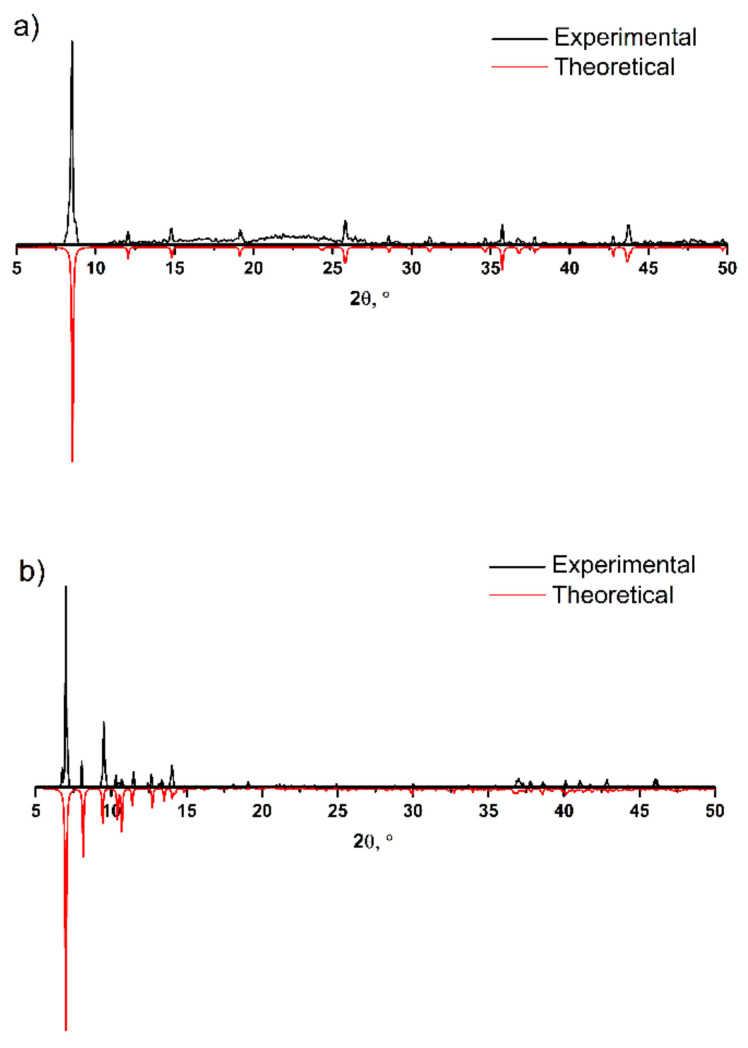
Experimental (top) vs calculated (bottom) powder diffraction patterns for compounds **4** (**a**) and **5** (**b**).

**Figure 2:**
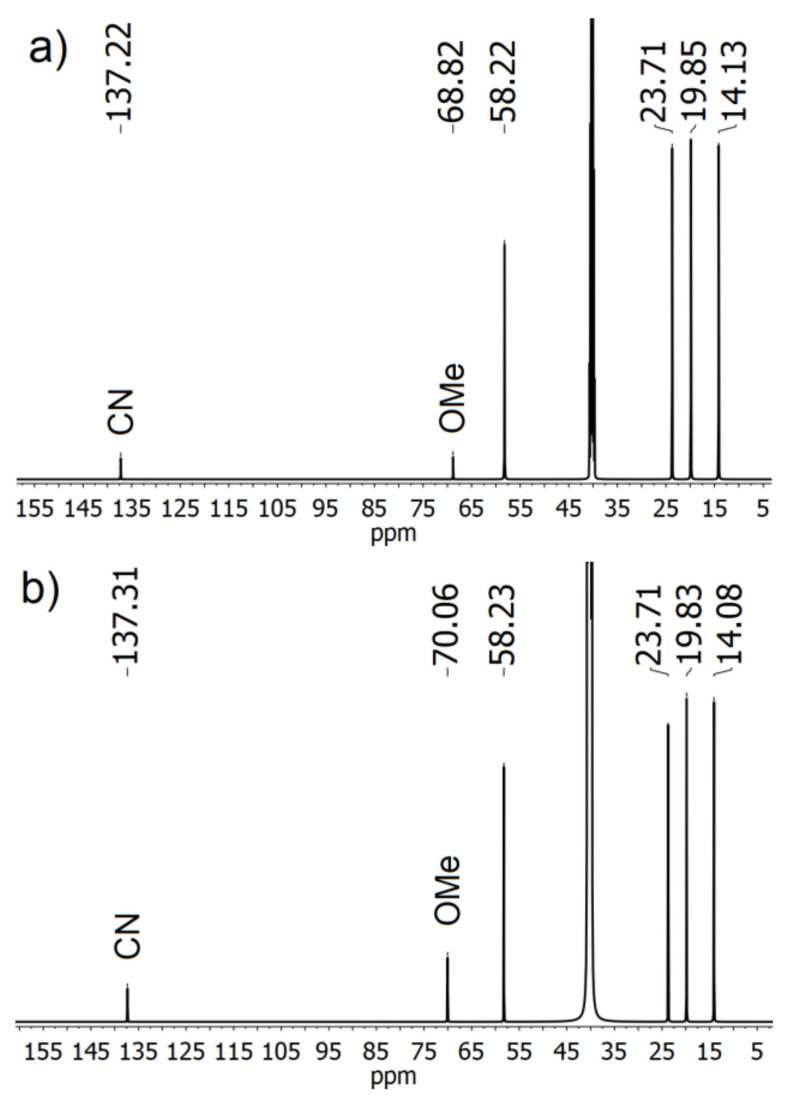
Experimental ^13^C-NMR spectra for compounds **4** (**a**) and **5** (**b**) in DMSO-d_6_ solution.

The second approach is the substitution of the terminal iodide ligands in the discrete cluster anions [{Mo_6_I_8_}I_6_]^2−^ and [{W_6_I_8_}I_6_]^2−^. These reactions were carried out under solvothermal conditions in methanol. In this case, orange-red solutions were formed, which, after cooling, were filtered from an excess of NaCN, mixed with an equal volume of H_2_O and evaporated. Addition of H_2_O was found to be necessary in order to prevent the formation of oil after concentration of solutions in pure methanol. The obtained red crystalline solids were isolated and investigated. The use of methanol in solvothermal synthesis made it possible to avoid the problem of hydrolysis of clusters at elevated temperatures. However, in this case, the methylate anion competes with cyanide, so that compounds based on apically heteroleptic anions *trans*-[Mo_6_I_8_(CN)_4_(MeO)_2_]^2−^ and *trans*-[W_6_I_8_(CN)_2_(MeO)_4_]^2−^ were obtained in high yields. The reaction products demonstrated phase purity (Figure 1), and the study of their solutions by ^13^C-NMR in d_6_-DMSO did not reveal the presence of geometric isomers or clusters with a different ratio of CN^−^ and MeO^−^ anions (Figure 2). Note that hexamolybdenum cluster complexes with methylate anions as apical ligands were described earlier [3], while similar W_6_ clusters are reported for the first time. Taking into account the high yields of reactions (86% and 60% for **4** and **5**, respectively), we can declare the successful preparation of heteroleptic cyanide complexes based on {Mo_6_I_8_}^4+^ and {W_6_I_8_}^4+^ cluster cores with the defined mutual orientation of CN^−^ and MeO^−^ ligands. This is of great importance not only for the chemistry of coordination polymers based on cluster complexes. Since methylate ligands are easily leaving ligands that are removed in the presence of acidic protons [3,34], synthesis of the new cluster anions opens the way to the design of new heteroleptic luminescent octahedral clusters of molybdenum and tungsten with predetermined geometries and charges through ligand exchange reactions.

### 2.2. Stability in Aqueous Solutions

The compounds based on anion [Mo_6_I_8_(CN)_6_]^2−^ belong to a small family of water-soluble molybdenum and tungsten halide clusters, which are the goal of many researches in the field [35,36]. There is also a challenge to prepare hydrolytically stable clusters of molybdenum and tungsten. To date, two examples of water-stable molybdenum cluster complexes have been reported, namely K_2_[{Mo_6_Br_8_}(CN)_6_] [31] and Na_2_[{Mo_6_I_8_}(OPOPh_2_)_6_] [37], and only one tungsten compound [{W_6_I_8_}(DMSO)_6_](NO_3_)_4_, which exists in aqueous solution for several days [38]. The latter complex exhibits phototoxicity, which is the reason for the high interest in such compounds.

**Figure 3:**
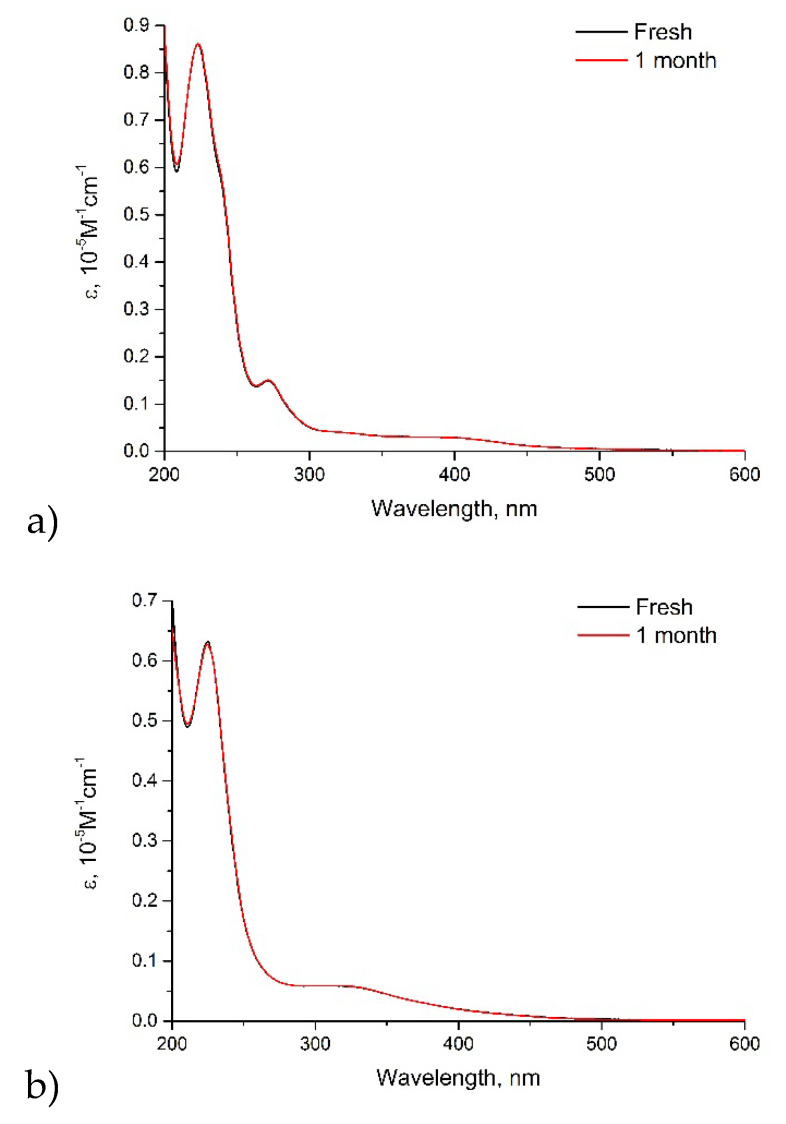
UV-Vis spectra of compounds **4** (**a**) and **5** (**b**) recorded for the freshly prepared aqueous solutions and for the solutions aged for 1 month.

Freshly prepared salts **4** and **5** (based on *trans*-[{Mo_6_I_8_}(CN)_4_(MeO)_2_]^2−^ and *trans*-[{W_6_I_8_}(CN)_2_(MeO)_4_]^2−^ anions, respectively) display good solubility in H_2_O, while dried samples of the compounds are soluble in aqueous methanol. We found that electronic absorption spectra of aerated aqueous or H_2_O solutions of compounds **1**–**5** showed invariability during long storage at room temperature (Figure 3). This indicates the stabilization of cluster molybdenum and tungsten halides upon coordination of CN^−^ ligands. Moreover, compounds **4** and **5** can be recrystallized from aqueous methanol showing no substitution of MeO^−^ ligands. ^13^C-NMR spectra for recrystallized samples match those for the freshly prepared compounds. Octahedral halide cluster complexes of molybdenum and tungsten usually have low hydrolytic stability because of replacement of the apical ligands by H_2_O or OH^−^ over time or hydrolysis of the cluster core itself [39]. Thus, the coordination of cyanide ligands significantly increased the hydrolytic stability of the clusters, which may open the way for investigation of their potential biological applications.

### 2.3. Crystal Structures

**Figure 4:**
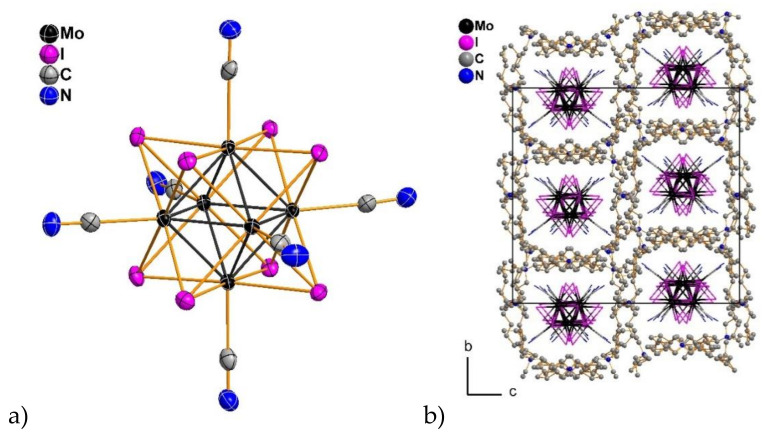
Structure of the [{Mo_6_I_8_}(CN)_6_]^2−^ cluster anion in the structure of compound **2**. Atoms are shown as thermal ellipsoids of 50% probability (**a**); packing of the cluster anions and Bu_4_N^+^ cations in the structure of compound **2**, view in *bc* plane (**b**).

Compound (Bu_4_N)_2_[{Mo_6_I_8_}(CN)_6_]·0.7H_2_O (**2**) crystallizes in monoclinic crystal system, *C*2/*c* space group. Asymmetric unit contains two [{Mo_6_I_8_}(CN)_6_]^2−^ cluster anions as well as four Bu_4_N^+^ cations with partially disordered CH_3_- groups and three partially occupied positions of solvate H_2_O molecules. The [{Mo_6_I_8_}(CN)_6_]^2−^ anion demonstrates a typical geometry of octahedral {Mo_6_(µ_3_-X_8_)} (X = halide) cluster core containing 24 cluster valence electrons (Figure 4a, Table 1). The average Mo-Mo and Mo-I bond distances (2.680(5) and 2.773(8) Å, respectively) agree well with the corresponding values observed for clusters with {Mo_6_I_8_}^4+^ core [40,41,42]. Each Mo atom of the cluster core is coordinated by apical CN^−^ ligand. The Mo-C distances display the average length of 2.188(8) Å, which is in the range of values that were reported for hexanuclear cyanide clusters of Mo [32,43,44]. The crystal packing includes the cluster anions forming a column along the a axis and Bu_4_N^+^ cations, which fill the space between anions forming a cationic sublattice with channel cavities (Figure 4b). The crystallographically observable lattice H_2_O molecules are located in proximity with cluster anions forming hydrogen bonds (2.96–3.07 Å) with N atoms of CN^−^ ligands.

Compound (Bu_4_N)_2_[{Mo_6_I_8_}(CN)_6_]·2DMSO (**3**) crystallizes in the triclinic crystal system, *P͞*1 space group. Asymmetric unit contains half of the cluster anions (three Mo atoms, four I atoms and atoms of three CN^−^ groups) as well as one Bu_4_N^+^ cation and one DMSO molecule (Figure S1). All positions are fully occupied. The structure of the cluster anion is similar to the one found in structure **2**. The crystal packing (Figure S2) is formed by the numerous interactions of hydrogen bond acceptors (O atoms of DMSO molecules and N atoms of CN^−^ groups) with -CH_2_- and CH_3_- groups of Bu_4_N^+^ cations and DMSO molecules. The corresponding -CH···O and -CH···N distances are within the range of 3.26–3.60 Å. In addition, weak hydrogen bonds were observed between -CH_3_ and -CH_2_- groups of Bu_4_N^+^ cation and µ_3_-I ligands of cluster anions. The C-I distances are 3.88 and 3.77 Å for -CH_3_ and -CH_2_- groups, respectively.

**Figure 5:**
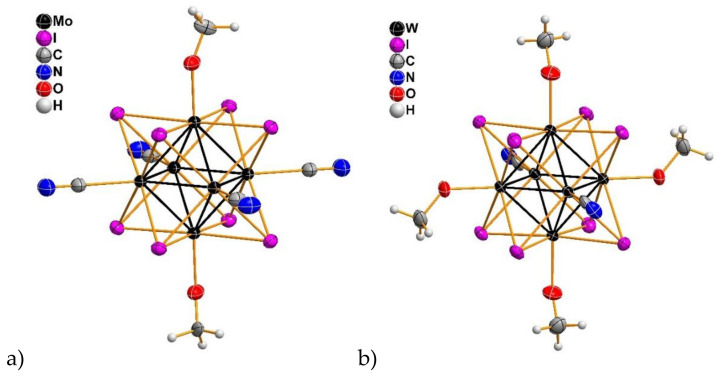
Structure of the [{Mo_6_I_8_}(CN)_4_(MeO)_2_]^2−^ cluster anions in the structure of compound **4** (**a**); structure of the [{W_6_I_8_}(CN)_2_(MeO)_4_]^2−^ cluster anion in the structure of compound **5** (**b**); atoms are shown as thermal ellipsoids of 50% probability.

Compound (Bu_4_N)_2_[{Mo_6_I_8_}(CN)_4_(MeO)_2_] (**4**) crystallizes in tetragonal crystal system, *I*4/*mmm* space group. Asymmetric unit contains two Mo atoms, one iodine atom, one CN^−^ group, one MeO^−^ group, and one N atom, four C atoms and 9 H atoms of Bu_4_N^+^ cation. Terminal -CH_3_ group of Bu_4_N^+^ cation is disordered over two equivalent positions. Terminal -CH_3_ group of MeO^−^ ligand is disordered over four positions by the 4-fold axis passing through the Mo and O atoms. All other positions are fully occupied. The *trans*-[{Mo_6_I_8_}(CN)_4_(MeO)_2_]^2−^ cluster anion (Figure 5a) displays the {Mo_6_I_8_}^4+^ cluster core, which is coordinated by four CN^−^ groups in the equatorial plane and two MeO^−^ groups in the *trans*-position. The corresponding Mo-C interatomic distances are 2.190(7) Å, and the Mo-O bonds are 2.049(8) Å. While the Mo-C distances match well the corresponding bond lengths found in other hexanuclear cyanide clusters of Mo, the Mo-O bonds are slightly shorter than ones reported previously for [Mo_6_I_8_(MeO)_6_]^2−^ anion (2.055–2.110 Å) [3]. In comparison with structure **2**, compound **4** demonstrates the different ways of packing cluster anions and Bu_4_N^+^ cations (Figure S3). Cluster anions are isolated in the center of pseudo-cubic cell formed by the alkyl groups of Bu_4_N^+^ cations.

Compound (Bu_4_N)_2_[{W_6_I_8_}(MeO)_4_(CN)_2_]·5H_2_O (**5**) crystallizes in a monoclinic crystal system, *P*2_1_/*n* space group. The asymmetric unit includes half of the *trans*-[{W_6_I_8_}(CN)_2_(MeO)_4_]^2−^ cluster anion (three W atoms, four I atoms, atoms of CN^−^ group, and two MeO^−^ ligands), one fully ordered Bu_4_N^+^ cation and four positions of O atoms of solvate H_2_O molecules, three of which are partially occupied. The cluster anion represent the 24-electron {W_6_I_8_}^4+^ core with a slightly distorted {W_6_} octahedron. The average W-W and W-I distances (2.671(8) and 2.82(1) Å, respectively) are comparable with the corresponding values found in hexanuclear tungsten iodides with various terminal ligands [1,2,34,45,46,47]. Two W atoms in the *trans*-position are coordinated by CN^−^ ligands, while four remaining W atoms in the equatorial plane are coordinated by a methylate anion (Figure 5b). The corresponding W-C and W-O distances are 2.195(7) and 2.048(5) Å, respectively. These bond lengths are similar to Mo-C bond lengths in the [Mo_6_I_8_(CN)_6_]^2−^ cluster anion (Table 1) and Mo-O bond lengths in the [{Mo_6_I_8_}(MeO)_6_]^2−^ anion.

The crystal packing of compound **5** (Figure S4) is formed by the cluster anions bound with solvate H_2_O molecules by a net of hydrogen bonds. Particularly, terminal N atoms of CN^−^ groups form OH···N bonds with a length of 3.0 Å, while the O atoms of MeO^−^ ligands form OH···O bonds with a length of 2.82–2.87 Å. Lengths of OH···O bonds between H_2_O molecules vary in the range of 2.80–2.99 Å. The Bu_4_N^+^ cations are included in the hydrogen-bonded framework of cluster anions and H_2_O molecules.

### 2.4. Calculated Geometry and Electronic Structure

**Figure 6:**
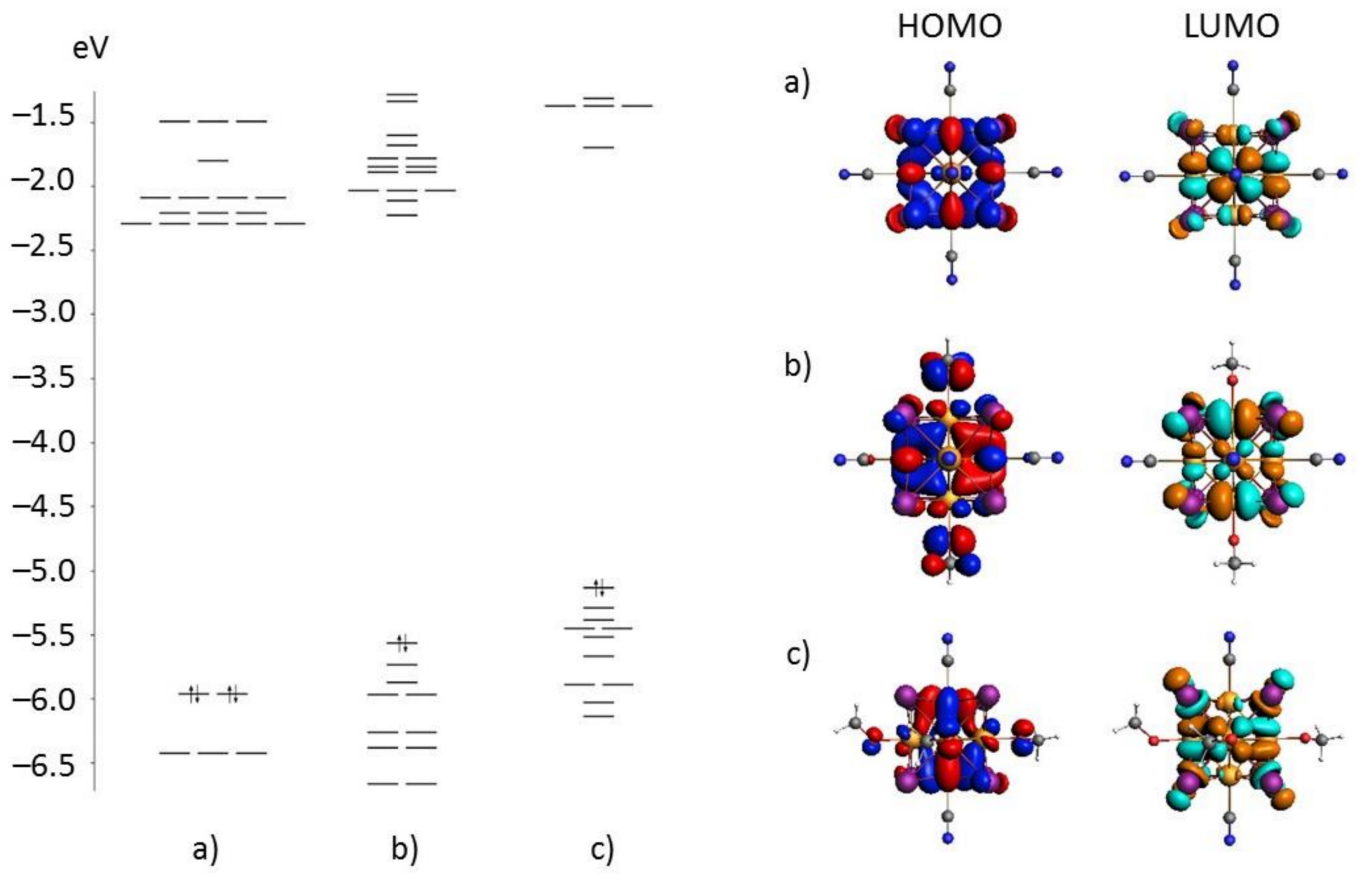
Left: energy level diagrams of the [{Mo_6_I_8_}(CN)_6_]^2−^, *trans*-[{Mo_6_I_8_}(CN)_4_(MeO)_2_]^2−^, and *trans*-[{W_6_I_8_}(CN)_2_(MeO)_4_]^2−^ cluster anions (**a**–**c**, respectively). Right: structure of the HOMOs and LUMOs of the [{Mo_6_I_8_}(CN)_6_]^2−^, *trans*-[{Mo_6_I_8_}(CN)_4_(MeO)_2_]^2−^, and *trans*-[{W_6_I_8_}(CN)_2_(MeO)_4_]^2−^ cluster anions (**a**–**c**, respectively).

Optimized interatomic distances for [{Mo_6_I_8_}(CN)_6_]^2−^, *trans*-[{Mo_6_I_8_}(CN)_4_(MeO)_2_]^2−^ and *trans*-[{W_6_I_8_}(CN)_2_(MeO)_4_]^2−^ cluster anions are close to the experimentally found values, although all calculated bond lengths tend to slightly increase (Table 2). Energy level diagrams in the near frontier region (Figure 6) show some differences between electronic structures of heteroligand clusters and [{Mo_6_I_8_}(CN)_6_]^2−^ anions. Frontier orbitals of the latter include almost degenerated HOMO and HOMO-1, which are localized primarily on the {Mo_6_I_8_}^4+^ core with a small contribution (~3%) of atomic orbitals of C and N atoms. In combination with cluster core-centered LUMO, electronic structure of this anion is typical for face-capped clusters of the [{M_6_X_8_}L_6_] type [48,49,50,51]. At the same time, HOMOs of *trans*-[{Mo_6_I_8_}(CN)_4_(MeO)_2_]^2−^ and *trans*-[{W_6_I_8_}(CN)_2_(MeO)_4_]^2−^ clusters include *p* orbitals of O and C atoms of terminal MeO^−^ ligands with the total contribution of about 10% for Mo cluster and 7% for the W cluster. HOMO-LUMO gaps are 3.67, 3.33, and 3.43 eV for [{Mo_6_I_8_}(CN)_6_]^2−^, *trans*-[{Mo_6_I_8_}(CN)_4_(MeO)_2_]^2−^, and *trans*-[{W_6_I_8_}(CN)_2_(MeO)_4_]^2−^ clusters, respectively.

### 2.5. Luminescence Properties

**Figure 7:**
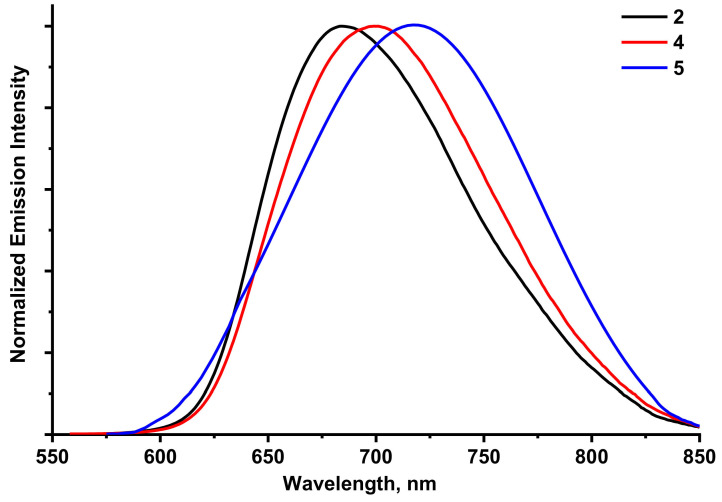
Normalized emission spectra of compounds **2**, **4** and **5** in acetonitrile solutions.

As noted above, 24-electron halide octahedral cluster complexes of molybdenum and tungsten demonstrate bright photoluminescence in the red region with microsecond lifetimes (phosphorescence) [1,3,6,16,37,40,42,52,53,54]. The spectroscopic and photophysical parameters, namely emission maximum wavelength λ_em_, lifetimes τ_em_ and quantum yields Φ_em_ for the deaerated acetonitrile solutions of compounds **2**, **4** and **5** as well as for the their precursors (Bu_4_N)_2_[{Mo_6_I_8_}I_6_] and (Bu_4_N)_2_[{W_6_I_8_}I_6_], are summarized in Table 3. All compounds showed typical broad emission bands with λ_em_ centered around 700 nm (Figure 7). Substitution of iodide apical ligands in [{Mo_6_I_8_}I_6_]^2−^ led to a blue-shift of the emission maxima, while transformation of [{W_6_I_8_}I_6_]^2−^ into [{W_6_I_8_}(CN)_2_(MeO)_4_]^2−^ resulted in a bathochromic shift of λ_em_. The emission spectral band shapes of [{W_6_I_8_}(CN)_2_(MeO)_4_]^2−^ is notably broader than the shapes of the {Mo_6_I_8_}^4+^-based complexes (Figure 7). Emission lifetimes of acetonitrile solutions of **2**, **4** and **5** are longer than those of corresponding [{M_6_I_8_}I_6_]^2−^ complexes, and τ_em_ values are comparable for hexamolybdenum anions in compounds **2** and **4** and higher than for the hexatungsten complex in **5** (Table 3). The relative emission quantum yields Φ_em_ greatly decrease in the row [Mo_6_I_8_(CN)_4_(MeO)_2_]^2−^ > [Mo_6_I_8_(CN)_6_]^2−^ > [W_6_I_8_(CN)_2_(MeO)_4_]^2−^. It is interesting to note that Φ_em_ values of deaerated acetonitrile solutions [{Mo_6_I_8_}(CN)_6_]^2−^ and [{W_6_I_8_}(CN)_2_(MeO)_4_]^2−^ are very low in comparison with those for the initial cluster complexes. At the same time, Φ_em_ of [Mo_6_I_8_(CN)_4_(MeO)_2_]^2−^ somewhat exceeds the quantum yield of [{Mo_6_I_8_}I_6_]^2−^.

## 3. Materials and Methods

Mo_6_I_12_ was synthesized by a high-temperature treatment of stoichiometric amounts of Mo and I_2_ at 700 °C in a sealed silica tube for 4 days [55]. (Bu_4_N)_2_[{Mo_6_I_8_}I_6_] was synthesized from Cs_2_[{Mo_6_I_8_}I_6_] following the reported procedure [55]. (Bu_4_N)_2_[{W_6_I_8_}I_6_] was synthesized using the reaction of W_6_Cl_12_ with a KI/LiI melt (70/30 mol%) followed by the dissolution of the cooled melt in ethanol and the precipitation of (Bu_4_N)_2_[{W_6_I_8_}I_6_] by adding Bu_4_NI to the solution [56,57]. Other reagents and solvents employed were commercially available and used as received without further purification.

Synthesis of compound **1** was carried out using a vibratory ball mill of the following construction: steel balls with a diameter of 3 mm (total mass 200 g) were charged into a cylindrical steel reactor (100 cm^3^ volume and 50 mm height) fitted with a flange cover. The frequency of the vertical reciprocating motion of the reactor was 100 Hz, the amplitude was 10 mm. Elemental (CHN) analysis was performed on a Euro EA3000 CHNS-O Analyzer (EuroVector, Pavia, Lombardy, Italy). Energy-dispersive X-ray spectroscopy (EDS) was performed on TM3000 TableTop Scanning Electron Microscope (Hitachi, Ltd., Marunouchi, Chiyoda-ku, Tokyo, Japan) with QUANTAX 70 EDS for SEM equipment (Bruker Corporation, Billerica, MA, USA). Infrared spectra were recorded with a Vertex 80 FT-IR spectrometer (Bruker Corporation, Billerica, MA, USA) in a KBr pellet. UV-Vis spectra were recorded in H_2_O with a Cary 60 spectrophotometer (Agilent Technologies, Inc., Santa Clara, California, USA) at room temperature in the range 200–600 nm. X-ray powder diffraction data were collected on a PW1820/1710 diffractometer (Philips, Amsterdam, Netherlands) (Cu Kα radiation, graphite monochromator, silicon plate used as an external standard). The thermogravimetric properties were studied using a TG 209 F1 Iris Thermo Microbalance (NETZSCH-Gerätebau GmbH, Selb, Germany) in the temperature range of 25–800 °C at a rate of 10°·min^−1^ in a He flow (30 mL·min^−1^). The ^13^C-NMR spectra were recorded from a DMSO-d_6_ solution at room temperature on Avance III 500 FT-spectrometer (Bruker Corporation, Billerica, MA, USA) with working frequency 125.73 MHz. The ^13^C-NMR chemical shifts are reported in ppm of the δ scale and referred to signals of the solvents (39.50 ppm). Excitation and emission photoluminescence spectra were recorded with a spectrofluorometer Fluorolog 3 spectrofluorometer (Horiba, Ltd., Kyoto, Japan) equipped with ozone-free 450 W Xe lamp, cooled R928/1860 photon detector (Hamamatsu Photonics K.K., Hamamatsu City, Shizuoka, Japan) with refrigerated chamber PC177CE-010 (Products for Research, Inc., Danvers, MA, USA) and double grating monochromators. Excitation and emission spectra were corrected for source intensity and detector spectral response by standard correction curves. The same instrument was used for determination emission lifetimes. The relative emission quantum yields (*Φ*_em_) for the deaerated acetonitrile solutions were estimated by using [Ru(bpy)_3_]Cl_2_ as a standard: *Φ*_em_ = 0.04 in aerated water [58].

### 3.1. Synthesis of Cs_1.3_Na_0.7_[{Mo_6_I_8_}(CN)_6_]·2H_2_O (**1**)

Mo_6_I_12_ (900 mg, 0.43 mmol) and NaCN (700 mg, 14.29 mmol) were placed in the vibratory ball mill. Mechanochemical activation was carried out during 12 h. The reaction products were dissolved in water (100 mL) and filtered forming clear orange solution. Solution of CsCl (200 mg, 1.19 mmol) in 5 mL of H_2_O was added, and then the solution was evaporated to a volume of about 10 mL. The forming red crystalline precipitate was filtered off and dried in air. Yield: 615 mg (73%). EDS: Cs:Na:Mo:I = 1.4:0.7:6:8.1. Anal. Calcd. for C_6_Cs_1.3_H_4_I_8_Mo_6_N_6_Na_0.7_O_2_: C 3.7, H 0.2, N 4.3. Found C 3.6, H 0.2, N 4.4%. UV-Vis (H_2_O): λ_max_, nm (ε, mol^−1^ dm^3^ cm^−1^) 215 (81834), 240 (81897), 272 (25126), 324* (sh, 5407), 391 (4516). IR (cm^−1^): ν(CN) 2118.

### 3.2. Synthesis of (Bu_4_N)_2_[{Mo_6_I_8_}(CN)_6_]·0.7H_2_O (**2**)

#### 3.2.1. Metathesis Reaction

Compound **1** (200 mg, 0.10 mmol) was dissolved in 10 mL of H_2_O, then a solution of Bu_4_NI (100 mg, 0.31 mmol) in 10 mL of H_2_O was added. The precipitate was separated by centrifugation, washed with hot water and dried in air. Yield: 180 mg (78%). EDS: Mo:I = 6.0:8.3. Anal. Calcd. for C_38_H_7__3.4_I_8_Mo_6_N_8_O_0.7_: C 20.3, H 3.3, N 5.0. Found C 20.5, H 3.3, N 5.1%. IR (cm^−1^): ν(CN) 2121. UV-Vis (CH_3_CN): λ_max_, nm (ε, mol^−1^ dm^3^ cm^−1^) 217 (74210), 242 (77041), 273 (22426), 332* (5443), 398 (4572).

#### 3.2.2. Solvothermal Ligand Exchange Reaction

A mixture of (Bu_4_N)_2_[{Mo_6_I_8_}I_6_] (150 mg, 0.05 mmol) and NaCN (150 mg, 3.06 mmol) were mixed with 3 mL of H_2_O in a glass tube. The tube was sealed, heated to 120 °C, held at this temperature for 24 h and then cooled to room temperature at a natural rate (about 50 °C/h). The reaction led to a dark brown insoluble powder and a pale yellow solution. The solution was filtered, evaporated to a volume of about 1 mL, cooled to the room temperature and kept in air. One day later, a few bright red needle crystals of compound **2** precipitated from the solution.

### 3.3. Synthesis of (Bu_4_N)_2_[{Mo_6_I_8_}(CN)_6_]·2DMSO (**3**)

Compound **2** (150 mg, 0.076 mmol) was dissolved in 2 mL of DMSO at a temperature of 80 °C. The solution was left at room temperature in a closed vial. Red crystals of compound **3** were precipitated for 12 h. Crystals were collected on a filter paper and dried in air. Yield: 100 mg (63%). EDS: Mo:I = 6.0:8.3. Anal. Calcd. for C_42_H_84_I_8_Mo_6_N_8_O_2_S_2_: C 21.1, H 3.5, N 4.7, S 2.7. Found: C 20.9, H 3.5, N 4.6, S 2.9%. IR (cm^−1^): ν(CN) 2115, ν(SO) 1035.

### 3.4. Preparation of (Bu_4_N)_2_[{Mo_6_I_8_}(CN)_4_(MeO)_2_] (**4**)

A mixture of (Bu_4_N)_2_[{Mo_6_I_8_}I_6_] (150 mg, 0.05 mmol) and NaCN (100 mg, 2.04 mmol) were mixed with 5 mL of MeOH in a glass tube. The tube was sealed, heated to 100 °C, held at this temperature for 12 h and then cooled to room temperature at a natural rate (about 50 °C/h). The bright red solution was filtered from excess of NaCN and 5 mL of H_2_O were added. The solution was evaporated to about 3 mL, cooled to the room temperature and kept in air. Two days later, dark red block crystals of compound **4** were precipitated. Yield: 102 mg (86%). EDS: Mo:I = 6.0:7.8. Anal. Calcd. for C_38_H_84_I_8_Mo_6_N_6_O_5_: C 19.9, H 3.7, N 3.7. Found: C 20.1, H 3.4, N 3.8%. IR (cm^−1^): ν(CN) 2110. UV-Vis (H_2_O): λ_max_, nm (ε, M^−1^ dm^3^ cm^−1^): 215 (86130), 272 (15060), 382 (3030). ^13^C-NMR (500 MHz, DMSO-d_6_): 14.16, 19.87, 23.72, 58.23 (Bu_4_N^+^), 39.91 (DMSO-d_6_), 69.26 (MeO), 137.19 (CN).

### 3.5. Preparation of (Bu_4_N)_2_[{W_6_I_8_}(CN)_2_(MeO)_4_]·5H_2_O (**5**)

Compound **5** was prepared as described for compound **4** using (Bu_4_N)_2_[{W_6_I_8_}I_6_] (150 mg, 0.045 mmol) and NaCN (100 mg, 2.04 mmol) as precursors. The yield of yellow block crystals of **5** was 77 mg (60%). EDS: W:I = 6.0:8.1. Anal. Calcd. for C_38_H_94_I_8_N_4_O_9_W_6_: C 15.9, H 3.3, N 2.0. Found: C 15.9, H 3.3, N 2.1%. IR (cm^−1^): ν(CN) 2112. UV-Vis (H_2_O): λ, nm (ε, M^−1^ dm^3^ cm^−1^): 225 (63290), 311 (5890). ^13^C-NMR (500 MHz, DMSO-d_6_): 14.08, 19.83, 23.71, 58.24 (Bu_4_N^+^), 39.91 (DMSO-d_6_), 70.06 (MeO), 137.31 (CN).

### 3.6. Single Crystal Diffraction Studies

Single crystals of compounds **2**–**5** were picked up directly from the reaction mixtures. Diffraction data for **2**–**4** were obtained on an Xcalibur diffractometer (Agilent Technologies, Inc., Santa Clara, California, USA) equipped with an area CCD AtlasS2 detector (Mo Kα, λ = 0.71073 Å, graphite monochromator, ω-scans). Integration, absorption correction, and determination of unit cell parameters were performed using the CrysAlisPro program package [59]. Single crystal XRD data for compound **5** were collected with a D8 Venture diffractometer (Bruker Corporation, Billerica, MA, USA) equipped with an area CMOS PHOTON III detector and IµS 3.0 source (Mo Kα, λ = 0.71073 Å, φ- and ω-scan). Absorption corrections were applied with the use of the SADABS program [60]. All measurements were conducted at 150 K. The structures were solved by a dual space algorithm (SHELXT) [61] and refined by the full-matrix least squares technique (SHELXL) [62] in the anisotropic approximation (except hydrogen atoms). Positions of hydrogen atoms of organic ligands were calculated geometrically and refined in the riding model. Hydrogen atoms of the water molecules were not located. The crystallographic data and details of the structure refinement are summarized in Table 4. Selected bond distances are listed in Table 1. CCDC 2042364–2042367 contain the crystallographic data for **2**–**5**, respectively. These data can be obtained free of charge from The Cambridge Crystallographic Data Centre via www.ccdc.cam.ac.uk/structures.

### 3.7. Computational Details

Density functional theory (DFT) calculations were carried out for [{Mo_6_I_8_}(CN)_6_]^2−^, *trans*-[{Mo_6_I_8_}(CN)_4_(MeO)_2_]^2−^ and *trans*-[{W_6_I_8_}(CN)_2_(MeO)_4_]^2−^ clusters in ADF2017 program package (ver. 2017.114, Software for Chemistry & Materials BV, Amsterdam, The Netherlands) [63,64]. Optimization of geometric parameters for the cluster anions in C_1_ symmetry was carried out using VWN + S12g dispersion corrected density functional [65,66,67] and the all-electron TZP basis set [68]. The calculated vibrational spectra contained no imaginary frequencies. Single point calculations of bonding energies and molecular orbitals with geometries from the VWN + S12g/TZP level of theory were carried out with the dispersion-corrected hybrid density functional S12h [67] and the all-electron TZP basis set. The zero-order regular approximation (ZORA) was used in all calculations to take into account the scalar relativistic effects [69]. The calculations were performed using the CH_3_CN environment effects, which were added with a Conductor-like Screening Model (COSMO) [70]. The selected calculated interatomic distances are presented in Table 2.

## 4. Conclusions

In summary, the new octahedral anionic complexes based on {Mo_6_I_8_}^4+^ and {W_6_I_8_}^4+^ cluster cores with apically homoleptic or heteroleptic coordination environment including ambidentate CN^−^ ligands were obtained. The [{Mo_6_I_8_}(CN)_6_]^2−^ cluster anion was synthesized by implementation of mechanochemical activation to the polymeric MoI_2_, while two heteroleptic anionic complexes *trans*-[{Mo_6_I_8_}(CN)_4_(MeO)_2_]^2−^ and *trans*-[{W_6_I_8_}(CN)_2_(MeO)_4_]^2−^ were synthesized using solvothermal method. Compounds based on the new anions were isolated as crystalline salts in preparative amounts. It was found that the compounds could be recrystallized from aqueous solutions and H_2_O/MeOH mixtures, showing excellent hydrolytic stability. Investigation of spectroscopic properties of the new compounds revealed the bright red luminescence with typical lifetimes of the order of 100 microseconds. The relative quantum yield for complex [{Mo_6_I_8_}(CN)_4_(MeO)_2_]^2−^ reached 0.14 in a deoxygenated acetonitrile solution. Note that this is the first report of the luminescence properties of cyanide octahedral clusters of molybdenum and tungsten. Owing to the combination of including ambidentate inert cyanide and labile methylate ligands, the heteroleptic anions can be used as functional building blocks for coordination polymers, as a basis for the directed synthesis of more complex heteroligand clusters, and as promising objects for phototoxicity studies.

## Figures and Tables

**Table 1:** Selected interatomic distances within [Mo_6_I_8_(CN)_6_]^2−^, *trans*-[Mo_6_I_8_(CN)_4_(MeO)_2_]^2−^ and *trans*-[W_6_I_8_(CN)_2_(MeO)_4_]^2−^ clusters (range/average, Å) in the structures of compounds **2**–**5**.

	(Bu_4_N)_2_[Mo_6_I_8_(CN)_6_]·H_2_O (2)	(Bu_4_N)_2_[Mo_6_I_8_(CN)_6_]·2DMSO (3)	(Bu_4_N)_2_[Mo_6_I_8_(CN)_4_(MeO)_2_] (4)	(Bu_4_N)_2_[W_6_I_8_(MeO)_4_(CN)_2_]·5H_2_O (5)
M–M	2.6738(8)–2.6904(8)/2.680(5)	2.6832(4)–2.6990(3)/2.689(6)	2.6799(7)–2.6829(8)/2.681(1)	2.6558(4)–2.6772(4)/2.6703(8)
M–I	2.7581(8)–2.7903(7)/2.773(8)	2.7679(4)–2.7907(3)/2.778(6)	2.7714(3)–2.7974(3)/2.78(1)	2.7998(5)–2.8388(5)/2.82(1)
M–C	2.177(8)–2.200(8)/2.188(8)	2.19(3)3–2.201(3)/2.196(4)	2.190(7)	2.195(7)
M–O	-	-	2.049(8)	2.049(5)–2.051(5)/2.050(1)

**Table 2:** Calculated interatomic distances within [Mo_6_I_8_(CN)_6_]^2−^, *trans*-[Mo_6_I_8_(CN)_4_(MeO)_2_]^2−^ and *trans*-[W_6_I_8_(CN)_2_(MeO)_4_]^2−^ clusters (range/average, Å).

	[Mo_6_I_8_(CN)_6_]^2−^	[Mo_6_I_8_(CN)_4_(MeO)_2_]^2−^	[W_6_I_8_(CN)_2_(MeO)_4_]^2−^
M–M	2.711	2.704–2.722/2.712(8)	2.692–2.707/2.699(4)
M–I	2.862	2.872–2.934/2.89(2)	2.912–2.992/2.95(3)
M–C	2.207	2.218	2.214
M–O	–	2.018	2.020

**Table 3:** Spectroscopic and photophysical parameters of compounds **2**, **4** and **5** in deaerated acetonitrile solutions (λ_ex_ = 360 nm) in comparison with those reported for (Bu_4_N)_2_[{Mo_6_I_8_}I_6_] and (Bu_4_N)_2_[{W_6_I_8_}I_6_].

Compound/Anion	Deaerated MeCN Solution		
	λ_max_, nm	Φ_em_	τ_em_, µs
2/[{Mo_6_I_8_}(CN)_6_]^2−^	685	0.02	162
4/[{Mo_6_I_8_}(CN)_4_(MeO)_2_]^2−^	700	0.14	151
5/[{W_6_I_8_}(CN)_2_(MeO)_4_]^2−^	720	0.01	92
(Bu_4_N)_2_[{Mo_6_I_8_}I_6_]/[{Mo_6_I_8_}I_6_]^2−^ [54]	730	0.12	90
(Bu_4_N)_2_[W_6_I_8_}I_6_]/[{W_6_I_8_}I_6_]^2−^ [54]	685	0.23	35

**Table 4:** Crystal data, data collection, and refinement parameters for **2**–**5**.

Compound	2	3	4	5
Chemical Formula	(Bu_4_N)_2_[Mo_6_I_8_(CN)_6_]·0.7H_2_O	(Bu_4_N)_2_[Mo_6_I_8_(CN)_6_]·2DMSO	(Bu_4_N)_2_[Mo_6_I_8_(CN)_4_(MeO)_2_]	(Bu_4_N)_2_[W_6_I_8_(MeO)_4_(CN)_2_]·5H_2_O
Empirical Formula	C_38_H_7__3.4_I_8_Mo_6_N_8_O_0.7_	C_42_H_84_I_8_Mo_6_N_8_O_2_S_2_	C_38_H_78_I_8_Mo_6_N_6_O_2_	C_38_H_94_I_8_N_4_O_9_W_6_
Formula Weight	2244.48	2388.13	2241.90	2859.39
Temperature (K)	140(2)	150(2)	150(2)	150(2)
Crystal Size (mm^3^)	0.18 × 0.10 × 0.09	0.15 × 0.14 × 0.07	0.16 × 0.15 × 0.08	0.12 × 0.05 × 0.05
Crystal System	Monoclinic	Triclinic	Tetragonal	Monoclinic
Space Group	*C*2/*c*	*P͞*1	*I*4/*mmm*	*P*2_1_/*n*
Z	16	1	2	2
a (Å)	37.2726(6)	10.7807(4)	14.6656(3)	9.8189(3)
b (Å)	24.8482(5)	11.4600(5)	14.6656(3)	15.4901(5)
c (Å)	28.2845(5)	15.2964(7)	14.5746(4)	21.8314(8)
Α (°)	90	92.784(4)	90	90
β (°)	111.997(2)	91.598(3)	90	97.512(1)
γ (°)	90	113.849(4)	90	90
V (Å^3^)	24288.9(8)	1724.1(1)	3134.7(2)	3292.0(2)
D_calcd._ (g cm^−3^)	2.455	2.300	2.375	2.895
μ (Mo K*α*) (mm^−1^)	5.303	4.737	5.137	14.235
θ range (°)	1.95 to 25.35	1.947 to 29.066	1.964 to 29.580	1.882 to 27.482
*h, k, l* index ranges	−43 ≤ *h* ≤ 44 −29 ≤ *k* ≤ 24 −34 ≤ *l* ≤ 22	−14 ≤ *h* ≤ 14 −15 ≤ *k* ≤ 14 −20 ≤ *l* ≤ 11	−20 ≤ *h* ≤ 15 −19 ≤ *k* ≤ 19 −14 ≤ *l* ≤ 19	−12 ≤ *h* ≤ 12 −20 ≤ *k* ≤ 20 −25 ≤ *l* ≤ 28
F(000)	16624	1116	2080	2560
Reflections Collected	57967	12899	8980	50844
Independent Reflections	22209 (R_int_ = 0.0314)	7839 (R_int_ = 0.0177)	1223 (R_int_ = 0.0139)	7549 (R_int_ = 0.0547)
Observed Reflections [I > 2σ(I)]	16412	6675	1101	6533
R Indices [I > 2σ(I)]	R_1_ = 0.0389 wR_2_ = 0.0864	R_1_ = 0.0215 wR_2_ = 0.0457	R_1_ = 0.0199 wR_2_ = 0.0553	R_1_ = 0.0306 wR_2_ = 0.0560
R Indices (all data)	R_1_ = 0.0643 wR_2_ = 0.0952	R_1_ = 0.0289 wR_2_ = 0.0479	R_1_ = 0.0237 wR_2_ = 0.0565	R_1_ = 0.0377 wR_2_ = 0.0591
GOOF on F^2^	1.053	0.992	1.091	1.042
Largest Diff. Peak and Hole (e Å^−3^)	1.745, −1.050	1.305, −0.568	2.815, −0.899	2.042, −1.951

## Supplementary Materials

The following are available online at. Figure S1: Fragment of structure of the compound **3** with numbered atoms (except hydrogens) of asymmetric unit. Atoms are shown as thermal ellipsoids of 50% probability; Figure S2: Packing of the cluster anions, Bu_4_N^+^ cations and DMSO molecules in the structure of compound **3**, view along *a* direction. Atoms are shown as thermal ellipsoids of 50% probability; Figure S3: Packing diagram for the compound **4**, view along *a* direction. Atoms are shown as thermal ellipsoids of 50% probability. Hydrogen atoms are not shown. Hydrogen atoms are not shown; Figure S4: Packing diagram for the compound **5**, view along *a* direction. Atoms are shown as thermal ellipsoids of 50% probability. Hydrogen atoms are not shown.
