# Supplementary material for: Cyanide Complexes Based on {Mo6I8}4+ and {W6I8}4+ Cluster Cores
Source: Molecules. 2020 Dec 8;25(24):5796. doi: 10.3390/molecules25245796 (PMC7764029; doi:10.3390/molecules25245796)

checkCIF/PLATON report

Structure factors have been supplied for datablock(s) pr771

THIS REPORT IS FOR GUIDANCE ONLY. IF USED AS PART OF A REVIEW PROCEDURE FOR PUBLICATION, IT SHOULD NOT REPLACE THE EXPERTISE OF AN EXPERIENCED CRYSTALLOGRAPHIC REFEREE.

No syntax errors found. CIF dictionary Interpreting this report

Datablock: pr771

```
Bond precision:  C-C = 0.0163 A          Wavelength=0.71073

Cell:           a=37.2726(6)    b=24.8482(5)        c=28.2845(5)
                alpha=90        beta=111.997(2)     gamma=90
Temperature:    140 K
```

	Calculated	Reported
Volume	24288.9(8)	24288.9(8)
Space group	C 2/c	C 2/c
Hall group	-C 2yc	-C 2yc
Moiety formula	2(C6 I8 Mo6 N6), C16 H32 N, C16 H29 N, C16 H30 N, C16 H36 N, 1.	?
Sum formula	C76 H127 I16 Mo12 N16 O1.40	C38 H73.40 I8 Mo6 N8 O0.70
Mr	4469.02	2244.48
Dx,g cm-3	2.444	2.455
Z	8	16
Mu (mm-1)	5.303	5.303
F000	16465.6	16624.0
F000’	16243.62	
h,k,lmax	44,29,34	44,29,34
Nref	22225	22210
Tmin,Tmax	0.535,0.620	0.903,1.000
Tmin’	0.381	

```
Correction method= # Reported T Limits: Tmin=0.903 Tmax=1.000
AbsCorr = MULTI-SCAN

Data completeness= 0.999          Theta(max)= 25.349

R(reflections)= 0.0393( 16412)    wR2(reflections)= 0.0979( 22210)

S = 1.053                   Npar= 1188
```

The following ALERTS were generated. Each ALERT has the format
test-name_ALERT_alert-type_alert-level.
Click on the hyperlinks for more details of the test.

Alert level B

```
PLAT315_ALERT_2_B Singly Bonded Carbon Detected (H-atoms Missing).       C335 Check
PLAT315_ALERT_2_B Singly Bonded Carbon Detected (H-atoms Missing).       C435 Check
PLAT360_ALERT_2_B Short  C(sp3)-C(sp3) Bond  C233     - C234     .       1.28 Ang.
PLAT410_ALERT_2_B Short Intra H...H Contact  H42A     ..H44D     .       1.85 Ang.
                                                      x,y,z  =      1_555 Check
```

Alert level C

```
PLAT041_ALERT_1_C Calc. and Reported SumFormula    Strings  Differ     Please Check
PLAT043_ALERT_1_C Calculated and Reported Mol. Weight Differ by ..      19.94 Check
PLAT068_ALERT_1_C Reported F000 Differs from Calcd (or Missing)...     Please Check
PLAT077_ALERT_4_C Unitcell Contains Non-integer Number of Atoms ..     Please Check
PLAT223_ALERT_4_C Solv./Anion  Resd 6  H   Ueq(max)/Ueq(min) Range        4.9 Ratio
PLAT234_ALERT_4_C Large Hirshfeld Difference N2       --C211     .       0.16 Ang.
PLAT234_ALERT_4_C Large Hirshfeld Difference N2       --C231     .       0.17 Ang.
PLAT234_ALERT_4_C Large Hirshfeld Difference C212     --C213     .       0.20 Ang.
PLAT234_ALERT_4_C Large Hirshfeld Difference C223     --C224     .       0.17 Ang.
PLAT234_ALERT_4_C Large Hirshfeld Difference C233     --C234     .       0.20 Ang.
PLAT234_ALERT_4_C Large Hirshfeld Difference C323     --C324     .       0.20 Ang.
PLAT234_ALERT_4_C Large Hirshfeld Difference C333     --C335     .       0.19 Ang.
PLAT234_ALERT_4_C Large Hirshfeld Difference C341     --C342     .       0.16 Ang.
PLAT234_ALERT_4_C Large Hirshfeld Difference C342     --C345     .       0.17 Ang.
PLAT234_ALERT_4_C Large Hirshfeld Difference C133     --C134     .       0.20 Ang.
PLAT241_ALERT_2_C High   MainMol Ueq as Compared to Neighbors of       C231 Check
PLAT241_ALERT_2_C High   MainMol Ueq as Compared to Neighbors of       C241 Check
PLAT242_ALERT_2_C Low    MainMol Ueq as Compared to Neighbors of         N2 Check
PLAT242_ALERT_2_C Low    MainMol Ueq as Compared to Neighbors of       C213 Check
PLAT242_ALERT_2_C Low    MainMol Ueq as Compared to Neighbors of         N3 Check
PLAT242_ALERT_2_C Low    MainMol Ueq as Compared to Neighbors of       C323 Check
PLAT250_ALERT_2_C Large U3/U1 Ratio for Average U(i,j) Tensor ....        2.5 Note
PLAT260_ALERT_2_C Large Average Ueq of Residue Including        N4      0.102 Check
PLAT342_ALERT_3_C Low Bond Precision on  C-C Bonds ..............    0.01631 Ang.
PLAT360_ALERT_2_C Short  C(sp3)-C(sp3) Bond  C231     - C232     .       1.41 Ang.
PLAT360_ALERT_2_C Short  C(sp3)-C(sp3) Bond  C413     - C414     .       1.43 Ang.
PLAT362_ALERT_2_C Short  C(sp3)-C(sp2) Bond  C442     - C443     .       1.40 Ang.
PLAT430_ALERT_2_C Short Inter D...A Contact  N12      ..O2W      .       2.88 Ang.
                                                      x,y,z  =      1_555 Check
PLAT906_ALERT_3_C Large K Value in the Analysis of Variance ......      3.703 Check
PLAT910_ALERT_3_C Missing # of FCF Reflection(s) Below Theta(Min).         10 Note
PLAT911_ALERT_3_C Missing FCF Refl Between Thmin & STh/L=    0.600          6 Report
PLAT971_ALERT_2_C Check Calcd Resid. Dens.  1.39A   From C233            1.70 eA-3
PLAT977_ALERT_2_C Check Negative Difference Density on H22E             -0.33 eA-3
PLAT977_ALERT_2_C Check Negative Difference Density on H31C             -0.37 eA-3
PLAT977_ALERT_2_C Check Negative Difference Density on H32G             -0.37 eA-3
PLAT977_ALERT_2_C Check Negative Difference Density on H44D             -0.32 eA-3
PLAT978_ALERT_2_C Number C-C Bonds with Positive Residual Density.          0 Info
```

Alert level G

```
FORMU01_ALERT_2_G  There is a discrepancy between the atom counts in the
            _chemical_formula_sum and the formula from the _atom_site* data.
            Atom count from _chemical_formula_sum:C38 H73.4 I8 Mo6 N8 O0.7
            Atom count from the _atom_site data:  C38 H63.5 I8 Mo6 N8 O0.7
CELLZ01_ALERT_1_G Difference between formula and atom_site contents detected.
```

CELLZ01_ALERT_1_G WARNING: H atoms missing from atom site list. Is this intentional?
From the CIF: _cell_formula_units_Z 16
From the CIF: _chemical_formula_sum C38 H73.40 I8 Mo6 N8 O0.70
TEST: Compare cell contents of formula and atom_site data

atom	Z*formula	cif sites	diff
C	608.00	608.00	0.00
H	1174.40	1016.00	158.40
I	128.00	128.00	0.00
Mo	96.00	96.00	0.00
N	128.00	128.00	0.00
O	11.20	11.20	0.00

PLAT002_ALERT_2_G Number of Distance or Angle Restraints on AtSite 27 Note
PLAT003_ALERT_2_G Number of Uiso or Uij Restrained non-H Atoms ... 54 Report
PLAT045_ALERT_1_G Calculated and Reported Z Differ by a Factor ... 0.50 Check
PLAT083_ALERT_2_G SHELXL Second Parameter in WGHT Unusually Large 122.30 Why ?
PLAT176_ALERT_4_G The CIF-Embedded .res File Contains SADI Records 2 Report
PLAT178_ALERT_4_G The CIF-Embedded .res File Contains SIMU Records 5 Report
PLAT186_ALERT_4_G The CIF-Embedded .res File Contains ISOR Records 1 Report
PLAT232_ALERT_2_G Hirshfeld Test Diff (M-X) I11 --Mo13 . 7.3 s.u.
PLAT232_ALERT_2_G Hirshfeld Test Diff (M-X) I12 --Mo12 . 6.0 s.u.
PLAT232_ALERT_2_G Hirshfeld Test Diff (M-X) I12 --Mo15 . 6.3 s.u.
PLAT232_ALERT_2_G Hirshfeld Test Diff (M-X) I12 --Mo16 . 6.8 s.u.
PLAT232_ALERT_2_G Hirshfeld Test Diff (M-X) I15 --Mo13 . 6.8 s.u.
PLAT232_ALERT_2_G Hirshfeld Test Diff (M-X) I17 --Mo12 . 5.3 s.u.
PLAT232_ALERT_2_G Hirshfeld Test Diff (M-X) I17 --Mo15 . 5.3 s.u.
PLAT232_ALERT_2_G Hirshfeld Test Diff (M-X) I18 --Mo14 . 5.0 s.u.
PLAT232_ALERT_2_G Hirshfeld Test Diff (M-X) I18 --Mo16 . 6.3 s.u.
PLAT232_ALERT_2_G Hirshfeld Test Diff (M-X) I21 --Mo23 . 5.0 s.u.
PLAT232_ALERT_2_G Hirshfeld Test Diff (M-X) I21 --Mo24 . 5.3 s.u.
PLAT232_ALERT_2_G Hirshfeld Test Diff (M-X) I22 --Mo25 . 5.3 s.u.
PLAT232_ALERT_2_G Hirshfeld Test Diff (M-X) I23 --Mo22 . 7.0 s.u.
PLAT232_ALERT_2_G Hirshfeld Test Diff (M-X) I26 --Mo21 . 6.5 s.u.
PLAT232_ALERT_2_G Hirshfeld Test Diff (M-X) I26 --Mo25 . 5.8 s.u.
PLAT232_ALERT_2_G Hirshfeld Test Diff (M-X) I28 --Mo22 . 5.8 s.u.
PLAT232_ALERT_2_G Hirshfeld Test Diff (M-X) I28 --Mo26 . 6.5 s.u.
PLAT300_ALERT_4_G Atom Site Occupancy of C224 Constrained at 0.7 Check
PLAT300_ALERT_4_G Atom Site Occupancy of C243 Constrained at 0.5 Check
PLAT300_ALERT_4_G Atom Site Occupancy of C244 Constrained at 0.5 Check
PLAT300_ALERT_4_G Atom Site Occupancy of C245 Constrained at 0.5 Check
PLAT300_ALERT_4_G Atom Site Occupancy of C246 Constrained at 0.5 Check
PLAT300_ALERT_4_G Atom Site Occupancy of C225 Constrained at 0.3 Check
PLAT300_ALERT_4_G Atom Site Occupancy of H22E Constrained at 0.7 Check
PLAT300_ALERT_4_G Atom Site Occupancy of H22F Constrained at 0.7 Check
PLAT300_ALERT_4_G Atom Site Occupancy of H22G Constrained at 0.7 Check
PLAT300_ALERT_4_G Atom Site Occupancy of H24C Constrained at 0.5 Check
PLAT300_ALERT_4_G Atom Site Occupancy of H24D Constrained at 0.5 Check
PLAT300_ALERT_4_G Atom Site Occupancy of H24E Constrained at 0.5 Check
PLAT300_ALERT_4_G Atom Site Occupancy of H24F Constrained at 0.5 Check
PLAT300_ALERT_4_G Atom Site Occupancy of H24G Constrained at 0.5 Check
PLAT300_ALERT_4_G Atom Site Occupancy of H24H Constrained at 0.5 Check
PLAT300_ALERT_4_G Atom Site Occupancy of H24I Constrained at 0.5 Check
PLAT300_ALERT_4_G Atom Site Occupancy of H24J Constrained at 0.5 Check
PLAT300_ALERT_4_G Atom Site Occupancy of H24K Constrained at 0.5 Check
PLAT300_ALERT_4_G Atom Site Occupancy of H24L Constrained at 0.5 Check
PLAT300_ALERT_4_G Atom Site Occupancy of H22H Constrained at 0.3 Check
PLAT300_ALERT_4_G Atom Site Occupancy of H22I Constrained at 0.3 Check
PLAT300_ALERT_4_G Atom Site Occupancy of H22J Constrained at 0.3 Check
PLAT300_ALERT_4_G Atom Site Occupancy of C333 Constrained at 0.7 Check
PLAT300_ALERT_4_G Atom Site Occupancy of C343 Constrained at 0.6 Check
PLAT300_ALERT_4_G Atom Site Occupancy of C344 Constrained at 0.6 Check
PLAT300_ALERT_4_G Atom Site Occupancy of C334 Constrained at 0.3 Check

```
PLAT300_ALERT_4_G Atom Site Occupancy of C345       Constrained at       0.4 Check
PLAT300_ALERT_4_G Atom Site Occupancy of C346       Constrained at       0.4 Check
PLAT300_ALERT_4_G Atom Site Occupancy of H33C       Constrained at       0.7 Check
PLAT300_ALERT_4_G Atom Site Occupancy of H33D       Constrained at       0.7 Check
PLAT300_ALERT_4_G Atom Site Occupancy of H34C       Constrained at       0.6 Check
PLAT300_ALERT_4_G Atom Site Occupancy of H34D       Constrained at       0.6 Check
PLAT300_ALERT_4_G Atom Site Occupancy of H34E       Constrained at       0.6 Check
PLAT300_ALERT_4_G Atom Site Occupancy of H34F       Constrained at       0.6 Check
PLAT300_ALERT_4_G Atom Site Occupancy of H34G       Constrained at       0.6 Check
PLAT300_ALERT_4_G Atom Site Occupancy of H33E       Constrained at       0.3 Check
PLAT300_ALERT_4_G Atom Site Occupancy of H33F       Constrained at       0.3 Check
PLAT300_ALERT_4_G Atom Site Occupancy of H34H       Constrained at       0.4 Check
PLAT300_ALERT_4_G Atom Site Occupancy of H34I       Constrained at       0.4 Check
PLAT300_ALERT_4_G Atom Site Occupancy of H34J       Constrained at       0.4 Check
PLAT300_ALERT_4_G Atom Site Occupancy of H34K       Constrained at       0.4 Check
PLAT300_ALERT_4_G Atom Site Occupancy of H34L       Constrained at       0.4 Check
PLAT300_ALERT_4_G Atom Site Occupancy of C433       Constrained at       0.5 Check
PLAT300_ALERT_4_G Atom Site Occupancy of C434       Constrained at       0.5 Check
PLAT300_ALERT_4_G Atom Site Occupancy of H43C       Constrained at       0.5 Check
PLAT300_ALERT_4_G Atom Site Occupancy of H43D       Constrained at       0.5 Check
PLAT300_ALERT_4_G Atom Site Occupancy of H43E       Constrained at       0.5 Check
PLAT300_ALERT_4_G Atom Site Occupancy of H43F       Constrained at       0.5 Check
PLAT300_ALERT_4_G Atom Site Occupancy of O1W        Constrained at       0.6 Check
PLAT300_ALERT_4_G Atom Site Occupancy of O2W        Constrained at       0.4 Check
PLAT300_ALERT_4_G Atom Site Occupancy of O3W        Constrained at       0.4 Check
PLAT302_ALERT_4_G Anion/Solvent/Minor-Residue Disorder (Resd  3  )       18% Note
PLAT302_ALERT_4_G Anion/Solvent/Minor-Residue Disorder (Resd  4  )       18% Note
PLAT302_ALERT_4_G Anion/Solvent/Minor-Residue Disorder (Resd  5  )        6% Note
PLAT302_ALERT_4_G Anion/Solvent/Minor-Residue Disorder (Resd  7  )      100% Note
PLAT302_ALERT_4_G Anion/Solvent/Minor-Residue Disorder (Resd  8  )      100% Note
PLAT302_ALERT_4_G Anion/Solvent/Minor-Residue Disorder (Resd  9  )      100% Note
PLAT304_ALERT_4_G Non-Integer Number of Atoms in ..... (Resd  7  )      0.60 Check
PLAT304_ALERT_4_G Non-Integer Number of Atoms in ..... (Resd  8  )      0.40 Check
PLAT304_ALERT_4_G Non-Integer Number of Atoms in ..... (Resd  9  )      0.40 Check
PLAT311_ALERT_2_G Isolated Disordered Oxygen Atom (No H's ?) .....       O1W Check
PLAT311_ALERT_2_G Isolated Disordered Oxygen Atom (No H's ?) .....       O2W Check
PLAT311_ALERT_2_G Isolated Disordered Oxygen Atom (No H's ?) .....       O3W Check
PLAT410_ALERT_2_G Short Intra H...H Contact  H43A     ..H43D     .      1.85 Ang.
                                                      x,y,z  =     1_555 Check
PLAT411_ALERT_2_G Short Inter H...H Contact  H33C     ..H443     .      2.03 Ang.
                                         1/2-x,-1/2+y,1/2-z  =     4_545 Check
PLAT412_ALERT_2_G Short Intra XH3 .. XHn     H22D     ..H22I     .      2.01 Ang.
                                                      x,y,z  =     1_555 Check
PLAT413_ALERT_2_G Short Inter XH3 .. XHn     H22D     ..H22G     .      2.12 Ang.
                                                1-x,y,1/2-z  =     2_655 Check
PLAT413_ALERT_2_G Short Inter XH3 .. XHn     H41E     ..H34J     .      1.87 Ang.
                                            1/2-x,1/2-y,1-z  =     7_556 Check
PLAT431_ALERT_2_G Short Inter HL..A Contact  I23      ..N14      .      3.29 Ang.
                                                      x,y,z  =     1_555 Check
PLAT431_ALERT_2_G Short Inter HL..A Contact  I24      ..N25      .      3.26 Ang.
                                                 -x,y,1/2-z  =     2_555 Check
PLAT432_ALERT_2_G Short Inter X...Y Contact  N16      ..C345            2.95 Ang.
                                          1/2-x,1/2+y,1/2-z  =     4_555 Check
PLAT432_ALERT_2_G Short Inter X...Y Contact  O3W      ..C422            2.98 Ang.
                                                      x,y,z  =     1_555 Check
PLAT860_ALERT_3_G Number of Least-Squares Restraints .............       619 Note
PLAT913_ALERT_3_G Missing # of Very Strong Reflections in FCF ....         1 Note
PLAT961_ALERT_5_G Dataset Contains no Negative Intensities .......    Please Check
```

---

0 **ALERT level A** = Most likely a serious problem - resolve or explain
4 **ALERT level B** = A potentially serious problem, consider carefully

```
 37 ALERT level C = Check. Ensure it is not caused by an omission or oversight
102 ALERT level G = General information/check it is not something unexpected

  6 ALERT type 1 CIF construction/syntax error, inconsistent or missing data
 55 ALERT type 2 Indicator that the structure model may be wrong or deficient
  6 ALERT type 3 Indicator that the structure quality may be low
 75 ALERT type 4 Improvement, methodology, query or suggestion
  1 ALERT type 5 Informative message, check
```

It is advisable to attempt to resolve as many as possible of the alerts in all categories. Often the minor alerts point to easily fixed oversights, errors and omissions in your CIF or refinement strategy, so attention to these fine details can be worthwhile. In order to resolve some of the more serious problems it may be necessary to carry out additional measurements or structure refinements. However, the purpose of your study may justify the reported deviations and the more serious of these should normally be commented upon in the discussion or experimental section of a paper or in the "special_details" fields of the CIF. checkCIF was carefully designed to identify outliers and unusual parameters, but every test has its limitations and alerts that are not important in a particular case may appear. Conversely, the absence of alerts does not guarantee there are no aspects of the results needing attention. It is up to the individual to critically assess their own results and, if necessary, seek expert advice.

Publication of your CIF in IUCr journals

A basic structural check has been run on your CIF. These basic checks will be run on all CIFs submitted for publication in IUCr journals (*Acta Crystallographica*, *Journal of Applied Crystallography*, *Journal of Synchrotron Radiation*); however, if you intend to submit to *Acta Crystallographica Section C* or *E* or *IUCrData*, you should make sure that full publication checks are run on the final version of your CIF prior to submission.

Publication of your CIF in other journals

Please refer to the *Notes for Authors* of the relevant journal for any special instructions relating to CIF submission.

PLATON version of 22/12/2019; check.def file version of 13/12/2019

Datablock pr771 - ellipsoid plot

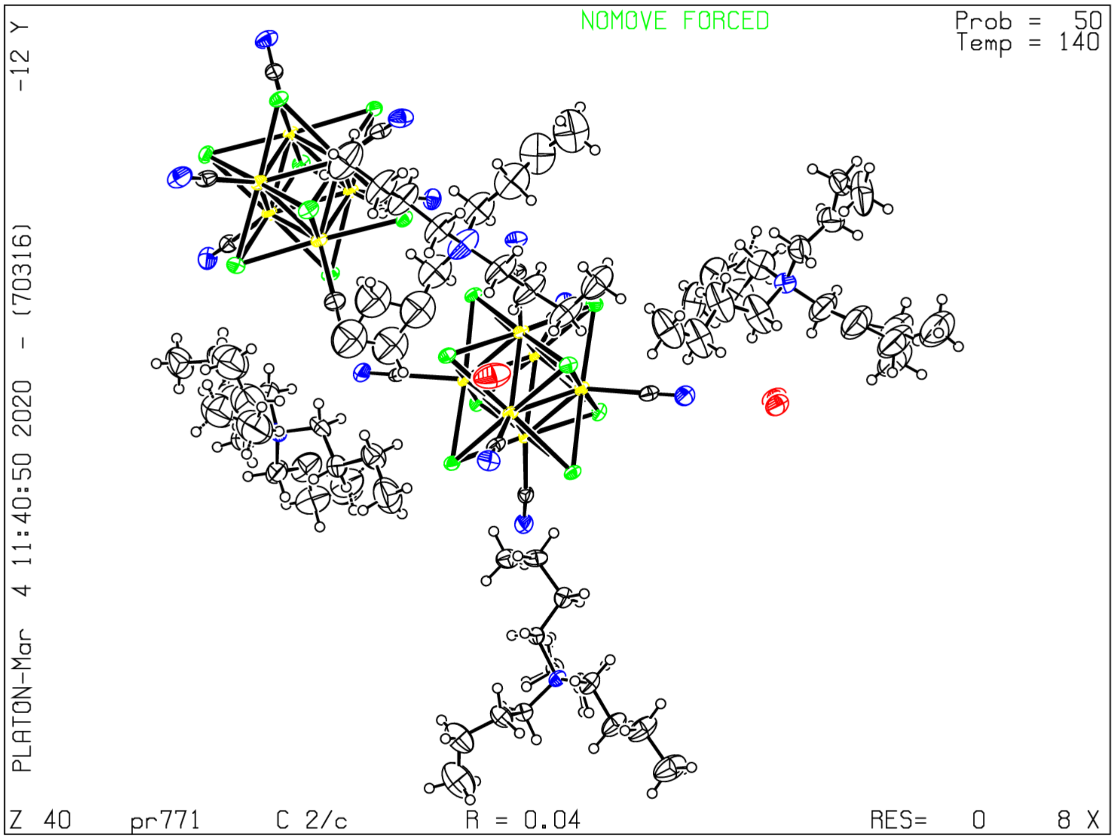

Supplement: Supplementary file 1 [file molecules-25-05796-s001.zip › Compound 2 checkcif.pdf]
