# Supplementary material for: Cyanide Complexes Based on {Mo6I8}4+ and {W6I8}4+ Cluster Cores
Source: Molecules. 2020 Dec 8;25(24):5796. doi: 10.3390/molecules25245796 (PMC7764029; doi:10.3390/molecules25245796)

checkCIF/PLATON report

Structure factors have been supplied for datablock(s) ys1831

THIS REPORT IS FOR GUIDANCE ONLY. IF USED AS PART OF A REVIEW PROCEDURE FOR PUBLICATION, IT SHOULD NOT REPLACE THE EXPERTISE OF AN EXPERIENCED CRYSTALLOGRAPHIC REFEREE.

No syntax errors found.   CIF dictionary   Interpreting this report

Datablock: ys1831

```
Bond precision:  C-C = 0.0045 A          Wavelength=0.71073

Cell:          a=10.7807(4)      b=11.4600(5)     c=15.2964(7)
               alpha=92.784(4)   beta=91.598(3)   gamma=113.849(4)
Temperature:   150 K
```

	Calculated	Reported
Volume	1724.08(14)	1724.08(14)
Space group	P -1	P -1
Hall group	-P 1	-P 1
Moiety formula	C6 I8 Mo6 N6, 2(C16 H36 N), 2(C2 H6 O S)	?
Sum formula	C42 H84 I8 Mo6 N8 O2 S2	C42 H84 I8 Mo6 N8 O2 S2
Mr	2388.13	2388.13
Dx,g cm-3	2.300	2.300
Z	1	1
Mu (mm-1)	4.737	4.737
F000	1116.0	1116.0
F000’	1102.39	
h,k,lmax	14,15,20	14,15,20
Nref	9243	7839
Tmin,Tmax	0.496,0.718	0.777,1.000
Tmin’	0.487	

```
Correction method= # Reported T Limits: Tmin=0.777 Tmax=1.000
AbsCorr = MULTI-SCAN

Data completeness= 0.848           Theta(max)= 29.066

R(reflections)= 0.0215( 6675)      wR2(reflections)= 0.0479( 7839)

S = 0.992                  Npar= 313
```

The following ALERTS were generated. Each ALERT has the format
test-name_ALERT_alert-type_alert-level.
Click on the hyperlinks for more details of the test.

Alert level C

```
PLAT094_ALERT_2_C Ratio of Maximum / Minimum Residual Density ....       2.30 Report
PLAT223_ALERT_4_C Solv./Anion  Resd 2  H   Ueq(max)/Ueq(min) Range        4.2 Ratio
PLAT601_ALERT_2_C Unit Cell Contains Solvent Accessible VOIDS of .         41 Ang**3
```

Alert level G

```
PLAT232_ALERT_2_G Hirshfeld Test Diff (M-X)  I1       --Mo3_a    .        9.0 s.u.
PLAT232_ALERT_2_G Hirshfeld Test Diff (M-X)  I2       --Mo1      .        6.4 s.u.
PLAT232_ALERT_2_G Hirshfeld Test Diff (M-X)  I2       --Mo2      .        7.0 s.u.
PLAT232_ALERT_2_G Hirshfeld Test Diff (M-X)  I2       --Mo3_a    .        7.1 s.u.
PLAT232_ALERT_2_G Hirshfeld Test Diff (M-X)  I3       --Mo1      .        9.2 s.u.
PLAT232_ALERT_2_G Hirshfeld Test Diff (M-X)  I3       --Mo3      .        6.9 s.u.
PLAT232_ALERT_2_G Hirshfeld Test Diff (M-X)  I3       --Mo2_a    .        6.6 s.u.
PLAT232_ALERT_2_G Hirshfeld Test Diff (M-X)  I4       --Mo1      .        8.9 s.u.
PLAT232_ALERT_2_G Hirshfeld Test Diff (M-X)  I4       --Mo2      .        7.6 s.u.
PLAT232_ALERT_2_G Hirshfeld Test Diff (M-X)  Mo1      --C1       .        6.2 s.u.
PLAT232_ALERT_2_G Hirshfeld Test Diff (M-X)  Mo2      --C2       .        6.5 s.u.
PLAT720_ALERT_4_G Number of Unusual/Non-Standard Labels ..........          6 Note
PLAT910_ALERT_3_G Missing # of FCF Reflection(s) Below Theta(Min).          1 Note
PLAT912_ALERT_4_G Missing # of FCF Reflections Above STh/L=  0.600       1404 Note
PLAT941_ALERT_3_G Average HKL Measurement Multiplicity ...........        1.6 Low
PLAT961_ALERT_5_G Dataset Contains no Negative Intensities .......     Please Check
PLAT978_ALERT_2_G Number C-C Bonds with Positive Residual Density.          0 Info
```

---

0 **ALERT level A** = Most likely a serious problem - resolve or explain
0 **ALERT level B** = A potentially serious problem, consider carefully
3 **ALERT level C** = Check. Ensure it is not caused by an omission or oversight
17 **ALERT level G** = General information/check it is not something unexpected

0 ALERT type 1 CIF construction/syntax error, inconsistent or missing data
14 ALERT type 2 Indicator that the structure model may be wrong or deficient
2 ALERT type 3 Indicator that the structure quality may be low
3 ALERT type 4 Improvement, methodology, query or suggestion
1 ALERT type 5 Informative message, check

It is advisable to attempt to resolve as many as possible of the alerts in all categories. Often the minor alerts point to easily fixed oversights, errors and omissions in your CIF or refinement strategy, so attention to these fine details can be worthwhile. In order to resolve some of the more serious problems it may be necessary to carry out additional measurements or structure refinements. However, the purpose of your study may justify the reported deviations and the more serious of these should normally be commented upon in the discussion or experimental section of a paper or in the "special_details" fields of the CIF. checkCIF was carefully designed to identify outliers and unusual parameters, but every test has its limitations and alerts that are not important in a particular case may appear. Conversely, the absence of alerts does not guarantee there are no aspects of the results needing attention. It is up to the individual to critically assess their own results and, if necessary, seek expert advice.

Publication of your CIF in IUCr journals

A basic structural check has been run on your CIF. These basic checks will be run on all CIFs submitted for publication in IUCr journals (*Acta Crystallographica*, *Journal of Applied Crystallography*, *Journal of Synchrotron Radiation*); however, if you intend to submit to *Acta Crystallographica Section C* or *E* or *IUCrData*, you should make sure that full publication checks are run on the final version of your CIF prior to submission.

Publication of your CIF in other journals

Please refer to the *Notes for Authors* of the relevant journal for any special instructions relating to CIF submission.

PLATON version of 18/09/2020; check.def file version of 20/08/2020

Datablock ys1831 - ellipsoid plot

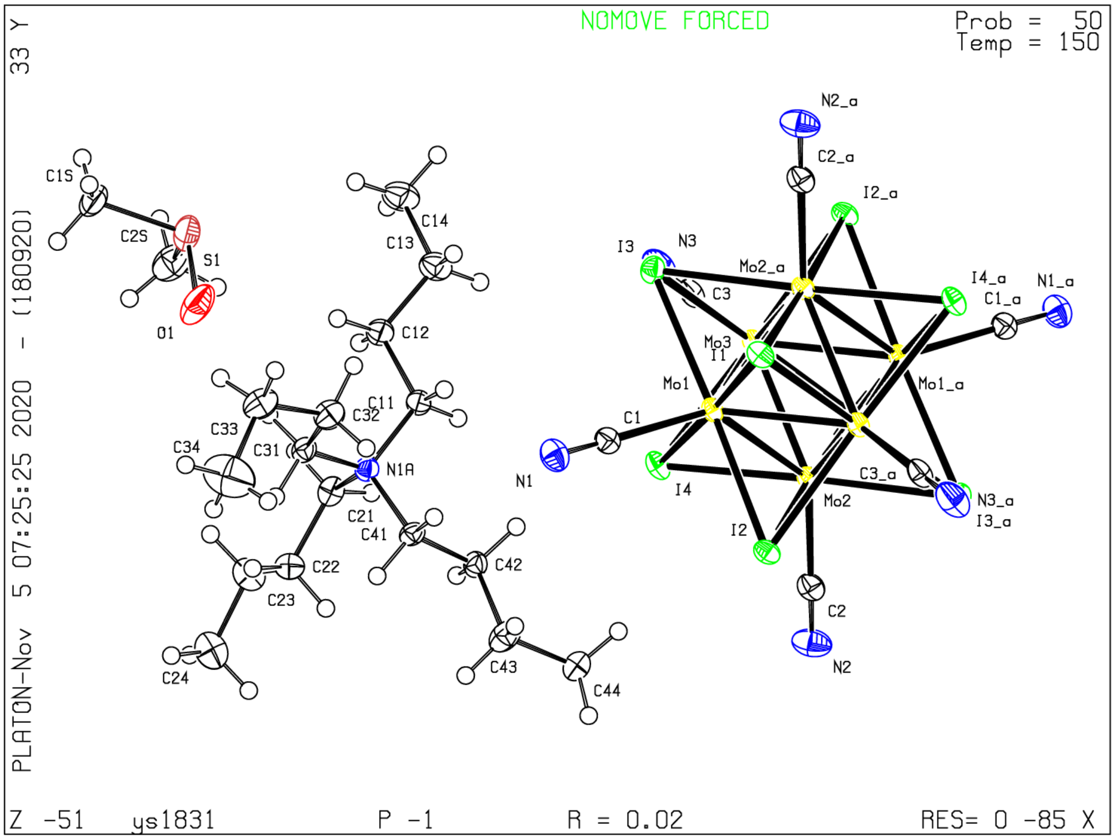

Supplement: Supplementary file 1 [file molecules-25-05796-s001.zip › Compound 3 checkcif.pdf]
