# Supplementary material for: Cyanide Complexes Based on {Mo6I8}4+ and {W6I8}4+ Cluster Cores
Source: Molecules. 2020 Dec 8;25(24):5796. doi: 10.3390/molecules25245796 (PMC7764029; doi:10.3390/molecules25245796)

# checkCIF/PLATON report

Structure factors have been supplied for datablock(s) pr780

THIS REPORT IS FOR GUIDANCE ONLY. IF USED AS PART OF A REVIEW PROCEDURE FOR PUBLICATION, IT SHOULD NOT REPLACE THE EXPERTISE OF AN EXPERIENCED CRYSTALLOGRAPHIC REFEREE.

No syntax errors found. CIF dictionary Interpreting this report

## Datablock: pr780

---

```
Bond precision:   C-C = 0.0080 A          Wavelength=0.71073

Cell:             a=14.6656(3)     b=14.6656(3)     c=14.5746(4)
                  alpha=90         beta=90          gamma=90
Temperature:      150 K
```

| | Calculated | Reported |
|---|---|---|
| Volume | 3134.70(15) | 3134.70(15) |
| Space group | I 4/m m m | I 4/m m m |
| Hall group | -I 4 2 | -I 4 2 |
| Moiety formula | C6 H6 I8 Mo6 N4 O2, 2(C16 H36 N) | ? |
| Sum formula | C38 H78 I8 Mo6 N6 O2 | C38 H78 I8 Mo6 N6 O2 |
| Mr | 2241.90 | 2241.90 |
| Dx,g cm-3 | 2.375 | 2.375 |
| Z | 2 | 2 |
| Mu (mm-1) | 5.137 | 5.137 |
| F000 | 2080.0 | 2080.0 |
| F000’ | 2052.27 | |
| h,k,lmax | 20,20,20 | 20,19,19 |
| Nref | 1294 | 1223 |
| Tmin,Tmax | 0.457,0.663 | 0.824,1.000 |
| Tmin’ | 0.423 | |

```
Correction method= # Reported T Limits: Tmin=0.824 Tmax=1.000
AbsCorr = MULTI-SCAN

Data completeness= 0.945          Theta(max)= 29.580

R(reflections)= 0.0199( 1101)     wR2(reflections)= 0.0565( 1223)

S = 1.091                   Npar= 72
```

---

The following ALERTS were generated. Each ALERT has the format
**test-name_ALERT_alert-type_alert-level**.
Click on the hyperlinks for more details of the test.

## Alert level B

```
PLAT971_ALERT_2_B Check Calcd Resid. Dens.  1.32A   From C2           3.00 eA-3
```

## Alert level C

```
PLAT094_ALERT_2_C Ratio of Maximum / Minimum Residual Density ....       3.13 Report
PLAT911_ALERT_3_C Missing FCF Refl Between Thmin & STh/L=    0.600          2 Report
```

## Alert level G

```
PLAT002_ALERT_2_G Number of Distance or Angle Restraints on AtSite          6 Note
PLAT003_ALERT_2_G Number of Uiso or Uij Restrained non-H Atoms ...          2 Report
PLAT083_ALERT_2_G SHELXL Second Parameter in WGHT  Unusually Large      13.16 Why ?
PLAT164_ALERT_4_G Nr. of Refined C-H H-Atoms in Heavy-Atom Struct.          3 Note
PLAT172_ALERT_4_G The CIF-Embedded .res File Contains DFIX Records          4 Report
PLAT178_ALERT_4_G The CIF-Embedded .res File Contains SIMU Records          1 Report
PLAT232_ALERT_2_G Hirshfeld Test Diff (M-X)  I2       --Mo1      .        7.5 s.u.
PLAT232_ALERT_2_G Hirshfeld Test Diff (M-X)  I2       --Mo2      .        9.0 s.u.
PLAT300_ALERT_4_G Atom Site Occupancy of O2         Constrained at       0.25 Check
PLAT300_ALERT_4_G Atom Site Occupancy of C2         Constrained at       0.25 Check
PLAT300_ALERT_4_G Atom Site Occupancy of H2A        Constrained at      0.125 Check
PLAT300_ALERT_4_G Atom Site Occupancy of H2B        Constrained at      0.125 Check
PLAT300_ALERT_4_G Atom Site Occupancy of H2C        Constrained at      0.125 Check
PLAT300_ALERT_4_G Atom Site Occupancy of C4N        Constrained at        0.5 Check
PLAT300_ALERT_4_G Atom Site Occupancy of H1N1       Constrained at        0.5 Check
PLAT300_ALERT_4_G Atom Site Occupancy of H1N2       Constrained at        0.5 Check
PLAT300_ALERT_4_G Atom Site Occupancy of H4N1       Constrained at        0.5 Check
PLAT300_ALERT_4_G Atom Site Occupancy of H4N2       Constrained at        0.5 Check
PLAT300_ALERT_4_G Atom Site Occupancy of H4N3       Constrained at        0.5 Check
PLAT300_ALERT_4_G Atom Site Occupancy of H32N       Constrained at        0.5 Check
PLAT301_ALERT_3_G Main Residue  Disorder ..............(Resd  1  )        27% Note
PLAT302_ALERT_4_G Anion/Solvent/Minor-Residue Disorder (Resd  2  )        24% Note
PLAT367_ALERT_2_G Long?  C(sp?)-C(sp?) Bond  C1N      - C2N      .       1.51 Ang.
PLAT367_ALERT_2_G Long?  C(sp?)-C(sp?) Bond  C2N      - C3N      .       1.54 Ang.
PLAT411_ALERT_2_G Short Inter H...H Contact  H31N     ..H2A      .       1.76 Ang.
                                                      x,y,z  =      1_555 Check
PLAT411_ALERT_2_G Short Inter H...H Contact  H31N     ..H2C      .       2.11 Ang.
                                                    1-y,x,z  =      3_655 Check
PLAT411_ALERT_2_G Short Inter H...H Contact  H31N     ..H2C      .       2.11 Ang.
                                                      y,x,z  =     24_555 Check
PLAT432_ALERT_2_G Short Inter X...Y Contact  C4N      ..C4N              2.99 Ang.
                                                      y,x,z  =     24_555 Check
PLAT720_ALERT_4_G Number of Unusual/Non-Standard Labels ..........          5 Note
PLAT779_ALERT_4_G Suspect or Irrelevant (Bond) Angle(s) in CIF . #        132 Check
              H2A  -C2   -H2C     1.555   1.555   3.655             19.30 Deg.
PLAT779_ALERT_4_G Suspect or Irrelevant (Bond) Angle(s) in CIF . #        133 Check
              H2A  -C2   -H2C    22.655   1.555  24.555             19.30 Deg.
PLAT779_ALERT_4_G Suspect or Irrelevant (Bond) Angle(s) in CIF . #        140 Check
              H2B  -C2   -H2B     2.665   1.555  23.665             19.20 Deg.
PLAT779_ALERT_4_G Suspect or Irrelevant (Bond) Angle(s) in CIF . #        141 Check
              H2B  -C2   -H2B     4.565   1.555  21.565             19.20 Deg.
PLAT779_ALERT_4_G Suspect or Irrelevant (Bond) Angle(s) in CIF . #        142 Check
              H2B  -C2   -H2B     1.555   1.555  21.565             25.60 Deg.
PLAT779_ALERT_4_G Suspect or Irrelevant (Bond) Angle(s) in CIF . #        143 Check
              H2B  -C2   -H2B     2.665   1.555  22.655             25.60 Deg.
PLAT779_ALERT_4_G Suspect or Irrelevant (Bond) Angle(s) in CIF . #        144 Check
```

```
             H2B  -C2   -H2B     4.565   1.555  23.665             36.30 Deg.
PLAT779_ALERT_4_G Suspect or Irrelevant (Bond) Angle(s) in CIF . #       145 Check
             H2B  -C2   -H2B     1.555   1.555   4.565             41.40 Deg.
PLAT779_ALERT_4_G Suspect or Irrelevant (Bond) Angle(s) in CIF . #       146 Check
             H2B  -C2   -H2B    22.655   1.555  23.665             41.40 Deg.
PLAT779_ALERT_4_G Suspect or Irrelevant (Bond) Angle(s) in CIF . #       180 Check
             H2C  -C2   -H2C     1.555   1.555  24.555             40.50 Deg.
PLAT779_ALERT_4_G Suspect or Irrelevant (Bond) Angle(s) in CIF . #       181 Check
             H2C  -C2   -H2C     3.655   1.555  22.655             40.50 Deg.
PLAT779_ALERT_4_G Suspect or Irrelevant (Bond) Angle(s) in CIF . #       207 Check
             C3N  -C2N  -C3N    22.655   1.555   1.555              0.00 Deg.
PLAT779_ALERT_4_G Suspect or Irrelevant (Bond) Angle(s) in CIF . #       213 Check
             C3N  -C3N  -C2N    22.655   1.555   1.555              0.00 Deg.
PLAT779_ALERT_4_G Suspect or Irrelevant (Bond) Angle(s) in CIF . #       214 Check
             C3N  -C3N  -H31N   22.655   1.555   1.555              0.00 Deg.
PLAT779_ALERT_4_G Suspect or Irrelevant (Bond) Angle(s) in CIF . #       215 Check
             C3N  -C3N  -H32N   22.655   1.555   1.555              0.00 Deg.
PLAT779_ALERT_4_G Suspect or Irrelevant (Bond) Angle(s) in CIF . #       216 Check
             C3N  -C3N  -H32N   22.655   1.555  22.655              0.00 Deg.
PLAT779_ALERT_4_G Suspect or Irrelevant (Bond) Angle(s) in CIF . #       217 Check
             C3N  -C3N  -C4N    22.655   1.555   1.555              0.00 Deg.
PLAT779_ALERT_4_G Suspect or Irrelevant (Bond) Angle(s) in CIF . #       218 Check
             C3N  -C3N  -C4N    22.655   1.555  22.655              0.00 Deg.
PLAT779_ALERT_4_G Suspect or Irrelevant (Bond) Angle(s) in CIF . #       224 Check
             C4N  -C3N  -H32N    1.555   1.555  22.655             23.00 Deg.
PLAT779_ALERT_4_G Suspect or Irrelevant (Bond) Angle(s) in CIF . #       225 Check
             C4N  -C3N  -H32N   22.655   1.555   1.555             23.00 Deg.
PLAT779_ALERT_4_G Suspect or Irrelevant (Bond) Angle(s) in CIF . #       229 Check
             C3N  -C4N  -C3N    22.655   1.555   1.555              0.00 Deg.
PLAT779_ALERT_4_G Suspect or Irrelevant (Bond) Angle(s) in CIF . #       230 Check
             C3N  -C4N  -H32N    1.555   1.555  22.655             27.00 Deg.
PLAT779_ALERT_4_G Suspect or Irrelevant (Bond) Angle(s) in CIF . #       231 Check
             C3N  -C4N  -H32N   22.655   1.555  22.655             27.00 Deg.
PLAT860_ALERT_3_G Number of Least-Squares Restraints .............        13 Note
PLAT912_ALERT_4_G Missing # of FCF Reflections Above STh/L=  0.600        64 Note
PLAT933_ALERT_2_G Number of OMIT Records in Embedded .res File ...         2 Note
PLAT961_ALERT_5_G Dataset Contains no Negative Intensities .......    Please Check
PLAT978_ALERT_2_G Number C-C Bonds with Positive Residual Density.         0 Info
```

---

  0 **ALERT level A** = Most likely a serious problem - resolve or explain
  1 **ALERT level B** = A potentially serious problem, consider carefully
  2 **ALERT level C** = Check. Ensure it is not caused by an omission or oversight
 57 **ALERT level G** = General information/check it is not something unexpected

  0 ALERT type 1 CIF construction/syntax error, inconsistent or missing data
 15 ALERT type 2 Indicator that the structure model may be wrong or deficient
  3 ALERT type 3 Indicator that the structure quality may be low
 41 ALERT type 4 Improvement, methodology, query or suggestion
  1 ALERT type 5 Informative message, check

---

It is advisable to attempt to resolve as many as possible of the alerts in all categories. Often the minor alerts point to easily fixed oversights, errors and omissions in your CIF or refinement strategy, so attention to these fine details can be worthwhile. In order to resolve some of the more serious problems it may be necessary to carry out additional measurements or structure refinements. However, the purpose of your study may justify the reported deviations and the more serious of these should normally be commented upon in the discussion or experimental section of a paper or in the "special_details" fields of the CIF. checkCIF was carefully designed to identify outliers and unusual parameters, but every test has its limitations and alerts that are not important in a particular case may appear. Conversely, the absence of alerts does not guarantee there are no aspects of the results needing attention. It is up to the individual to critically assess their own results and, if necessary, seek expert advice.

**Publication of your CIF in IUCr journals**

A basic structural check has been run on your CIF. These basic checks will be run on all CIFs submitted for publication in IUCr journals (*Acta Crystallographica*, *Journal of Applied Crystallography*, *Journal of Synchrotron Radiation*); however, if you intend to submit to *Acta Crystallographica Section C* or *E* or *IUCrData*, you should make sure that full publication checks are run on the final version of your CIF prior to submission.

**Publication of your CIF in other journals**

Please refer to the *Notes for Authors* of the relevant journal for any special instructions relating to CIF submission.

---

**PLATON version of 10/08/2020; check.def file version of 06/08/2020**

**Datablock pr780** - ellipsoid plot

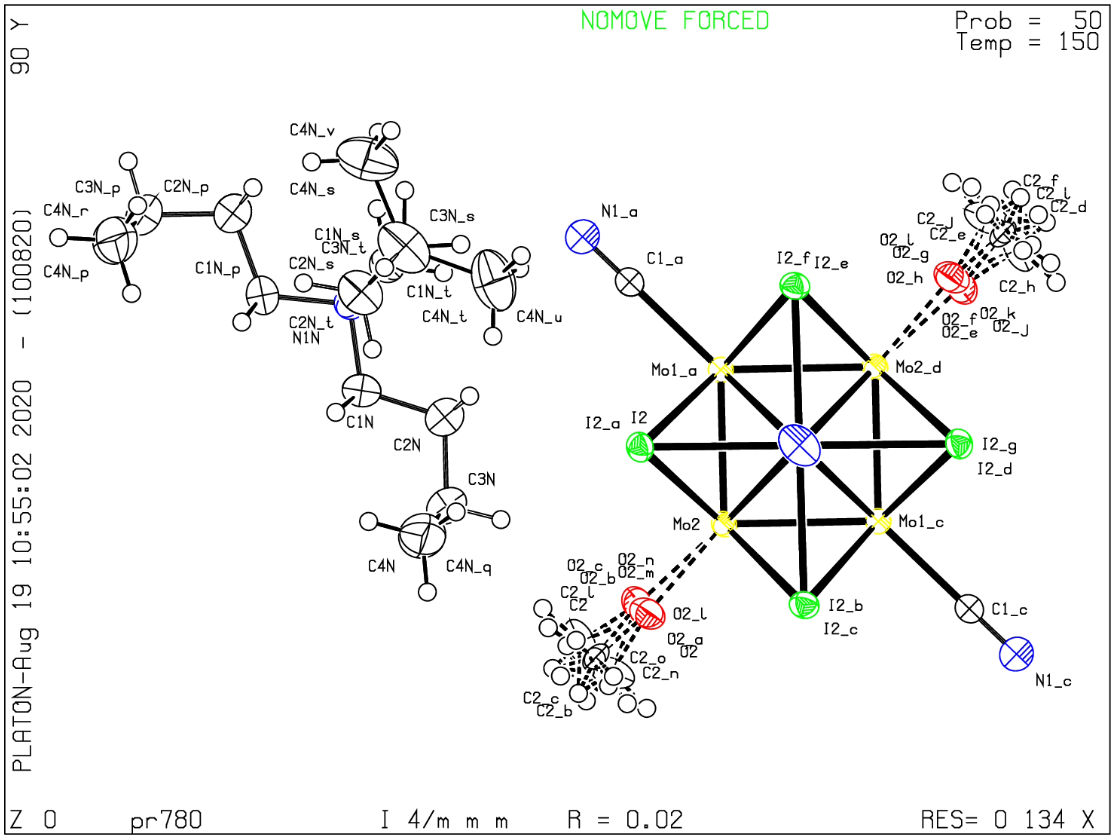

Supplement: Supplementary file 1 [file molecules-25-05796-s001.zip › Compound 4 checkcif.pdf]
