# Supplementary material for: Cyanide Complexes Based on {Mo6I8}4+ and {W6I8}4+ Cluster Cores
Source: Molecules. 2020 Dec 8;25(24):5796. doi: 10.3390/molecules25245796 (PMC7764029; doi:10.3390/molecules25245796)

checkCIF/PLATON report

Structure factors have been supplied for datablock(s) pr892

THIS REPORT IS FOR GUIDANCE ONLY. IF USED AS PART OF A REVIEW PROCEDURE FOR PUBLICATION, IT SHOULD NOT REPLACE THE EXPERTISE OF AN EXPERIENCED CRYSTALLOGRAPHIC REFEREE.

No syntax errors found. CIF dictionary Interpreting this report

Datablock: pr892

```
Bond precision:   C-C = 0.0101 A          Wavelength=0.71073

Cell:             a=9.8189(3)     b=15.4901(5)       c=21.8314(8)
                  alpha=90        beta=97.512(1)     gamma=90
Temperature:      150 K

                 Calculated                  Reported
Volume           3291.97(19)                 3291.97(19)
Space group      P 21/n                      P 21/n
Hall group       -P 2yn                      -P 2yn
                 2(C6 H12 I8 N2 O4 W6),
Moiety formula   4(C16 H36 N), 4(O0.75),     ?
                 7(O)
Sum formula      C76 H168 I16 N8 O18 W12     C38 H94 I8 N4 O9 W6
Mr               5718.67                     2869.47
Dx,g cm-3        2.885                       2.895
Z                1                           2
Mu (mm-1)        14.235                      14.235
F000             2560.0                      2580.0
F000'            2544.48
h,k,lmax         12,20,28                    12,20,28
Nref             7555                        7549
Tmin,Tmax        0.430,0.491                 0.590,0.746
Tmin'            0.179

Correction method= # Reported T Limits: Tmin=0.590 Tmax=0.746
AbsCorr = MULTI-SCAN

Data completeness= 0.999           Theta(max)= 27.482

R(reflections)= 0.0308( 6533)      wR2(reflections)= 0.0604( 7549)

S = 1.046                   Npar= 313
```

The following ALERTS were generated. Each ALERT has the format
test-name_ALERT_alert-type_alert-level.
Click on the hyperlinks for more details of the test.

Alert level B

```
PLAT306_ALERT_2_B Isolated Oxygen Atom (H-atoms Missing ?) .......        O1W Check
PLAT430_ALERT_2_B Short Inter D...A Contact  O1W      ..O2W      .       2.80 Ang.
                                                    x,y,z  =      1_555 Check
PLAT430_ALERT_2_B Short Inter D...A Contact  O1W      ..O3       .       2.82 Ang.
                                                    x,y,z  =      1_555 Check
```

Alert level C

```
PLAT041_ALERT_1_C Calc. and Reported SumFormula    Strings  Differ     Please Check
PLAT043_ALERT_1_C Calculated and Reported Mol. Weight Differ by ..      20.27 Check
PLAT068_ALERT_1_C Reported F000 Differs from Calcd (or Missing)...     Please Check
PLAT241_ALERT_2_C High   'MainMol' Ueq as Compared to Neighbors of         O2 Check
PLAT342_ALERT_3_C Low Bond Precision on  C-C Bonds ...............    0.01008 Ang.
PLAT430_ALERT_2_C Short Inter D...A Contact  O1W      ..O2       .       2.87 Ang.
                                                 -1+x,y,z  =      1_455 Check
PLAT911_ALERT_3_C Missing FCF Refl Between Thmin & STh/L=    0.600          4 Report
PLAT971_ALERT_2_C Check Calcd Resid. Dens.  0.75A   From I2              2.37 eA-3
PLAT971_ALERT_2_C Check Calcd Resid. Dens.  0.73A   From W2              1.93 eA-3
PLAT971_ALERT_2_C Check Calcd Resid. Dens.  0.69A   From I4              1.81 eA-3
PLAT972_ALERT_2_C Check Calcd Resid. Dens.  0.70A   From I2             -2.00 eA-3
PLAT972_ALERT_2_C Check Calcd Resid. Dens.  0.73A   From I4             -1.84 eA-3
PLAT972_ALERT_2_C Check Calcd Resid. Dens.  0.65A   From W2             -1.77 eA-3
PLAT975_ALERT_2_C Check Calcd Resid. Dens.  0.73A   From O2W             1.13 eA-3
PLAT975_ALERT_2_C Check Calcd Resid. Dens.  0.63A   From O2W             1.08 eA-3
PLAT975_ALERT_2_C Check Calcd Resid. Dens.  0.54A   From O2W             0.99 eA-3
PLAT975_ALERT_2_C Check Calcd Resid. Dens.  0.65A   From O2W             0.90 eA-3
PLAT975_ALERT_2_C Check Calcd Resid. Dens.  0.69A   From O2W             0.78 eA-3
PLAT975_ALERT_2_C Check Calcd Resid. Dens.  0.83A   From O1W             0.72 eA-3
PLAT976_ALERT_2_C Check Calcd Resid. Dens.  0.81A   From O2             -0.69 eA-3
PLAT977_ALERT_2_C Check Negative Difference Density on H2B              -0.32 eA-3
PLAT977_ALERT_2_C Check Negative Difference Density on H23A             -0.32 eA-3
```

Alert level G

```
FORMU01_ALERT_2_G  There is a discrepancy between the atom counts in the
            _chemical_formula_sum and the formula from the _atom_site* data.
            Atom count from _chemical_formula_sum:C38 H94 I8 N4 O9 W6
            Atom count from the _atom_site data:  C38 H84 I8 N4 O9 W6
CELLZ01_ALERT_1_G Difference between formula and atom_site contents detected.
CELLZ01_ALERT_1_G WARNING: H atoms missing from atom site list. Is this intentional?
           From the CIF: _cell_formula_units_Z    2
           From the CIF: _chemical_formula_sum  C38 H94 I8 N4 O9 W6
           TEST: Compare cell contents of formula and atom_site data

           atom    Z*formula  cif sites diff
           C         76.00     76.00    0.00
           H        188.00    168.00   20.00
           I         16.00     16.00    0.00
           N          8.00      8.00    0.00
           O         18.00     18.00    0.00
           W         12.00     12.00    0.00
PLAT003_ALERT_2_G Number of Uiso or Uij Restrained non-H Atoms ...          2 Report
PLAT045_ALERT_1_G Calculated and Reported Z Differ by a Factor ...       0.50 Check
PLAT083_ALERT_2_G SHELXL Second Parameter in WGHT  Unusually Large      23.96 Why ?
PLAT178_ALERT_4_G The CIF-Embedded .res File Contains SIMU Records          1 Report
```

```
PLAT232_ALERT_2_G Hirshfeld Test Diff (M-X)  W1       --I1       .        5.5 s.u.
PLAT232_ALERT_2_G Hirshfeld Test Diff (M-X)  W1       --I4_a     .        5.3 s.u.
PLAT232_ALERT_2_G Hirshfeld Test Diff (M-X)  W2       --I1       .       20.5 s.u.
PLAT232_ALERT_2_G Hirshfeld Test Diff (M-X)  W2       --I2       .       15.0 s.u.
PLAT232_ALERT_2_G Hirshfeld Test Diff (M-X)  W3       --I3       .        5.5 s.u.
PLAT232_ALERT_2_G Hirshfeld Test Diff (M-X)  W3       --I2_a     .        7.3 s.u.
PLAT232_ALERT_2_G Hirshfeld Test Diff (M-X)  W3       --I4_a     .        7.3 s.u.
PLAT300_ALERT_4_G Atom Site Occupancy of O3W        Constrained at        0.5 Check
PLAT300_ALERT_4_G Atom Site Occupancy of O4W        Constrained at       0.25 Check
PLAT300_ALERT_4_G Atom Site Occupancy of O2W        Constrained at       0.75 Check
PLAT302_ALERT_4_G Anion/Solvent/Minor-Residue Disorder (Resd  3  )       100% Note
PLAT302_ALERT_4_G Anion/Solvent/Minor-Residue Disorder (Resd  5  )       100% Note
PLAT304_ALERT_4_G Non-Integer Number of Atoms in ..... (Resd  3  )       0.75 Check
PLAT304_ALERT_4_G Non-Integer Number of Atoms in ..... (Resd  5  )       0.75 Check
PLAT311_ALERT_2_G Isolated Disordered Oxygen Atom (No H's ?) .....        O2W Check
PLAT790_ALERT_4_G Centre of Gravity not Within Unit Cell: Resd.  #          5 Note
              O
PLAT860_ALERT_3_G Number of Least-Squares Restraints .............          6 Note
PLAT910_ALERT_3_G Missing # of FCF Reflection(s) Below Theta(Min).          1 Note
PLAT912_ALERT_4_G Missing # of FCF Reflections Above STh/L=  0.600          1 Note
PLAT913_ALERT_3_G Missing # of Very Strong Reflections in FCF ....          1 Note
PLAT933_ALERT_2_G Number of OMIT Records in Embedded .res File ...          4 Note
PLAT961_ALERT_5_G Dataset Contains no Negative Intensities .......     Please Check
PLAT978_ALERT_2_G Number C-C Bonds with Positive Residual Density.          0 Info
```

---

0 **ALERT level A** = Most likely a serious problem - resolve or explain
3 **ALERT level B** = A potentially serious problem, consider carefully
22 **ALERT level C** = Check. Ensure it is not caused by an omission or oversight
30 **ALERT level G** = General information/check it is not something unexpected

6 ALERT type 1 CIF construction/syntax error, inconsistent or missing data
33 ALERT type 2 Indicator that the structure model may be wrong or deficient
5 ALERT type 3 Indicator that the structure quality may be low
10 ALERT type 4 Improvement, methodology, query or suggestion
1 ALERT type 5 Informative message, check

It is advisable to attempt to resolve as many as possible of the alerts in all categories. Often the minor alerts point to easily fixed oversights, errors and omissions in your CIF or refinement strategy, so attention to these fine details can be worthwhile. In order to resolve some of the more serious problems it may be necessary to carry out additional measurements or structure refinements. However, the purpose of your study may justify the reported deviations and the more serious of these should normally be commented upon in the discussion or experimental section of a paper or in the "special_details" fields of the CIF. checkCIF was carefully designed to identify outliers and unusual parameters, but every test has its limitations and alerts that are not important in a particular case may appear. Conversely, the absence of alerts does not guarantee there are no aspects of the results needing attention. It is up to the individual to critically assess their own results and, if necessary, seek expert advice.

Publication of your CIF in IUCr journals

A basic structural check has been run on your CIF. These basic checks will be run on all CIFs submitted for publication in IUCr journals (*Acta Crystallographica*, *Journal of Applied Crystallography*, *Journal of Synchrotron Radiation*); however, if you intend to submit to *Acta Crystallographica Section C* or *E* or *IUCrData*, you should make sure that full publication checks are run on the final version of your CIF prior to submission.

Publication of your CIF in other journals

Please refer to the *Notes for Authors* of the relevant journal for any special instructions relating to CIF submission.

PLATON version of 10/08/2020; check.def file version of 06/08/2020

Datablock pr892 - ellipsoid plot

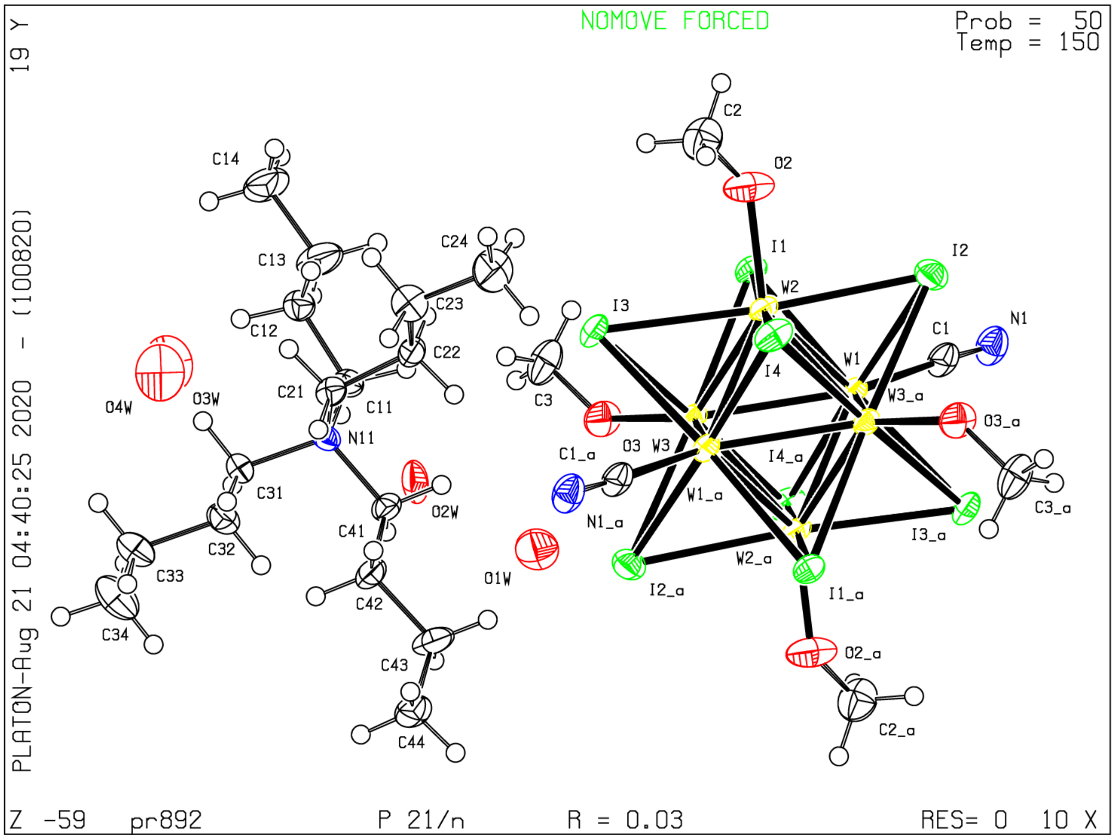

Supplement: Supplementary file 1 [file molecules-25-05796-s001.zip › Compound 5 checkcif.pdf]
